# Population genomic structure of Eurasian and African foxtail millet landrace accessions inferred from genotyping‐by‐sequencing

**DOI:** 10.1002/tpg2.20081

**Published:** 2021-02-04

**Authors:** Harriet V. Hunt, Natalia A. S. Przelomska, Michael G. Campana, James Cockram, H. Frances J. Bligh, Catherine J. Kneale, Olga I. Romanova, Elena V. Malinovskaya, Martin K. Jones

**Affiliations:** ^1^ McDonald Institute for Archaeological Research University of Cambridge Downing Street Cambridge CB2 3ER UK; ^2^ Comparative Plant and Fungal Biology Royal Botanic Gardens Kew Richmond TW9 3AE UK; ^3^ Department of Anthropology National Museum of Natural History Smithsonian Institution Washington DC 20560 USA; ^4^ Center for Conservation Genomics Smithsonian's National Zoo and Conservation Biology Institute Smithsonian Institution Washington DC 20008 USA; ^5^ Department of Archaeology University of Cambridge Downing Street Cambridge CB2 3DZ UK; ^6^ The John Bingham Laboratory NIAB 93 Lawrence Weaver Road Cambridge CB3 0LE UK; ^7^ Unilever R and D Colworth Park, Sharnbrook Bedfordshire MK44 1LQ UK; ^8^ N.I. Vavilov Institute of Plant Genetic Resources (VIR) St. Petersburg 190000 Russia

## Abstract

Foxtail millet [*Setaria italica* (L.) P. Beauv.] is the second most important millet species globally and is adapted to cultivation in diverse environments. Like its wild progenitor, green foxtail [*S. viridis* (L.) P. Beauv.], it is a model species for C4 photosynthetic pathways and stress tolerance genes in related bioenergy crops. We addressed questions regarding the evolution and spread of foxtail millet through a population genomic study of landraces from across its cultivated range in Europe, Asia, and Africa. We sought to determine population genomic structure and the relationship of domesticated lineages relative to green foxtail. Further, we aimed to identify genes involved in environmental stress tolerance that have undergone differential selection between geographical and genetic groups. Foxtail millet landrace accessions (*n* = 328) and green foxtail accessions (*n* = 12) were sequenced by genotyping‐by‐sequencing (GBS). After filtering, 5,677 single nucleotide polymorphisms (SNPs) were retained for the combined foxtail millet−green foxtail dataset and 5,020 for the foxtail millet dataset. We extended geographic coverage of green foxtail by including previously published GBS sequence tags, yielding a 4,515‐SNP dataset for phylogenetic reconstruction. All foxtail millet samples were monophyletic relative to green foxtail, suggesting a single origin of foxtail millet, although no group of foxtail millet was clearly the most ancestral. Four genetic clusters were found within foxtail millet, each with a distinctive geographical distribution. These results, together with archaeobotanical evidence, suggest plausible routes of spread of foxtail millet. Selection scans identified nine candidate genes potentially involved in environmental adaptations, particularly to novel climates encountered, as domesticated foxtail millet spread to new altitudes and latitudes.

## Core Ideas

- Foxtail millet is monophyletic with respect to its wild ancestor, green foxtail.
- There are four major genetic clusters in domestic foxtail.
- Domesticated foxtail likely adapted to novel environments as it spread from China.
- Genomic resource base for foxtail enhanced by wide geographic sample coverage.

**Abbreviations**
*F*_ST_
: fixation index
GBS: genotyping‐by‐sequencing
HKSV: green foxtail dataset in Huang et al., 2014
LD: linkage disequilibrium
PCA: principal components analysis
SNP: single nucleotide polymorphism
VIR: N.I. Vavilov Institute of Plant Genetic Resources.

## 1 INTRODUCTION

Foxtail millet [*Setaria italica* (L.) P. Beauv.] is one of the oldest domesticated cereals, cultivated from as early as 10,500 cal BP in northern China (Yang et al., 2012). It has recently been the subject of increased research interest, including the publication of multiple reference‐quality genome assemblies in 2012 (Bennetzen et al., 2012; Zhang et al., 2012). Its small diploid genome (515 Mb; 2*n* = 18), short generation time, and inbreeding nature make foxtail millet and its wild progenitor green foxtail valuable genetic models for C4 photosynthesis and abiotic and biotic stress tolerance in related crops within the Paniceae subfamily. *Setaria* is also a genomic model for related bioenergy crops with larger genomes, including switchgrass (*Panicum virgatum* L.), *Miscanthus* spp., Napiergrass [*Pennisetum purpureum* (Schumach.) Morrone], and sugarcane (*Saccharum officinarum* L.) (Brutnell, Bennetzen, & Vogel, 2015; Muthamilarasan & Prasad, 2015). To date, the foxtail millet genome sequence has facilitated the identification of adaptive genes and evolutionary dynamics including genes adaptive to drought and salt stress (J. Li, Dong, Li, Pan, & Yu, 2017; Liu et al., 2016; Tang et al., 2017), candidate genes in the C4 photosynthesis pathway (Huang, Studer, Schnable, Kellogg, & Brutnell, 2016), dynamics of genome evolution through transposon activity (Bennetzen, 2017), genes controlling leaf color (Li et al., 2016), pathogen resistance genes (Andersen & Nepal, 2017; Zhao et al., 2016), control of plant architecture and branching (Mauro‐Herrera & Doust, 2016; Mauro‐Herrera et al., 2013), and flowering time loci (Doust, Mauro‐Herrera, Hodge, & Stromski, 2017; Mauro‐Herrera et al., 2013).

In global terms, foxtail millet is economically the second most important millet {behind pearl millet, [*Cenchrus americanus* (L.) Morrone]; Upadhyaya et al., 2015}, yielding 6 Tg of grain annually (Goron & Raizada, 2015). Compared with other millets, its cultivation is exceptionally widespread in both tropical and temperate regions, and foxtail millet is grown on all the world's cultivable continents for grain, forage, or bird feed. This observation highlights the adaptation of foxtail millet to diverse agronomic environments, which vary in photoperiod, temperature, and seasonal water availability. However, the earliest archaeobotanical evidence for the exploitation of foxtail millet is from semi‐arid northern China (Lu et al., 2009; Yang et al., 2012). It became the dominant crop in this region in the Middle Neolithic and early Bronze Age periods, outstripping broomcorn millet (*Panicum miliaceum* L. subsp. *miliaceum*) in importance (Barton et al., 2009; Jia et al., 2016; Lee, Crawford, Liu, & Chen, 2007). Over subsequent millennia, the cultivation of foxtail millet was spread by people into north‐ and southwestern China, the Korean peninsula, Japan, Taiwan, southeast Asia, south and central Asia, southwest Asia, and Europe (Stevens et al., 2016). Today, the majority of its cultivation for human consumption is in China and India. The chronology of spread into different geographical regions is summarized by Stevens et al. (2016), but reevaluation and refinement through more systematic archaeobotanical sampling, direct dating, and clarification of identification criteria is needed in some areas.

The population genomic structure of foxtail millet landrace accessions (varieties assumed to have a long history of cultivation in a particular locality) can contribute to understanding its globalization process by identifying the geographical distribution of genetic groups and relating these to ecophysiology and archaeobotanical data. The aim of the current study is to provide a geographically comprehensive survey of genome‐wide single nucleotide polymorphism (SNP) variation in foxtail millet to infer the history of its domestication from the wild ancestor, green foxtail, spread, and adaptation to different environments. Two previous studies have analyzed SNP diversity in foxtail millet using low‐coverage whole‐genome resequencing (Jia et al., 2013) and genotyping‐by‐sequencing (GBS) approaches respectively (Upadhyaya et al., 2015). The sampled accessions in these studies were strongly biased toward collections from China and India, and neither study focused primarily on the geographical distribution of genomic variation.

Based on 328 landrace accessions, our study presents the most geographically balanced picture of genomic variation in Old World foxtail millet to date. It includes much denser coverage of accessions in Europe and Central Asia, which have been poorly represented in previous work. Of special significance was the inclusion of 119 accessions from Vavilov's collections held by the N.I. Vavilov Institute of Plant Genetic Resources (VIR), enabling coverage of Russia, the Caucasus, and central Asia. The VIR holds ∼4,750 foxtail millet accessions, making it the world's third largest collection (Goron & Raizada, 2015), but these accessions do not yet appear in the VIR's digital database (http://db.vir.nw.ru/virdb/maindb; last accessed 4 Oct. 2019), meaning that they have been largely overlooked in published studies.

We hypothesized that differentiation of each subpopulation from the ancestral group would have involved adaptation to the new ecological conditions encountered during the spread of domesticated foxtail millet. Archaeobotanical evidence favors the domestication of foxtail millet in the semi‐arid zones of northern China (Zhao, 2011), where it is a summer‐season crop that flowers in response to shortening days. From knowledge of selective pressures in other cultivated cereal species, key traits selected were most likely to have included those adapting plants to lower and higher latitudes and higher altitudes encountered en route to new areas of cultivation (Allaby et al., 2015). Such traits include flowering time response in nonnative daylength and seasonal regimes (X. Liu et al., 2017) and increased hardiness in higher altitudes, characterized, for example, by thicker stems and leaves, defense against ultraviolet (UV) radiation, dwarf stature, denser trichomes, and earlier flowering time (Kubota et al., 2015).

## 2 MATERIALS AND METHODS

### 2.1 Plant materials

The *Setaria* sample set comprised 359 foxtail millet individuals from 341 accessions and 21 green foxtail individuals from 21 accessions. These were obtained from five gene banks (VIR; National Institute of Agrobiological Sciences, Japan; International Crops Research Institute for the Semi‐Arid Tropics, India; Leibniz Institute of Plant Genetics and Crop Plant Research, Gatersleben, Germany; and USDA–ARS) (Supplemental Table S1). Sampling primarily used one individual per accession, with the exception of 18 accessions of foxtail millet that were represented by two individuals in the original sample set. These were filtered following sequencing as described below.

**Core Ideas**

- Foxtail millet is monophyletic with respect to its wild ancestor, green foxtail.
- There are four major genetic clusters in domestic foxtail.
- Domesticated foxtail likely adapted to novel environments as it spread from China.
- Genomic resource base for foxtail enhanced by wide geographic sample coverage.

### 2.2 GBS library preparation and sequencing and data processing

Leaf tissue from young seedlings was sampled, freeze‐dried, and used for DNA extraction, using a DNeasy Plant Mini Kit (Qiagen), following the manufacturer's protocol. DNA was checked for high molecular weight quality by electrophoresis on TAE‐agarose gels and quantified using a Qubit 2.0 fluorometer (ThermoFisher Scientific). Four 96‐plex libraries, each of 95 *Setaria* samples and one negative water control, were prepared for GBS using a modified protocol of Elshire et al. (2011) using *Pst*I as the restriction enzyme. Paired‐end sequencing of each library, giving 2 × 100 bp paired‐end reads, was performed on an Illumina HiSeq 2000 at the Centre for Genomic Research, University of Liverpool. The Stacks pipeline (version 1.40) was used to process the read data to call genotypes for each sample (Catchen, Hohenlohe, Bassham, Amores, & Cresko, 2013). The ‘process_radtags’ module was used for demultiplexing, barcode checking, and restriction enzyme cut‐site checking, as well as data quality control. The demultiplexed reads were all cut to 92 bp and then mapped to the foxtail millet cultivar Yugu 1 genome v 2.1 (Bennetzen et al., 2012) using bowtie2 (Langmead & Salzberg, 2012). The ‘ref_map’ module was run with a minimum stack depth of three to construct the catalog from which sets of loci and SNPs were output using various combinations of parameters in the ‘populations’ module. Following an initial run of ‘populations,’ the SNP set was imported into PLINK 1.9 (http://pngu.mgh.harvard.edu/purcell/plink/; (Purcell et al., 2007) and samples with large amounts of missing data were excluded using a filtering criterion of genotyping at 70% or more of the loci. We also excluded samples representing duplicate individuals for a single accession, those whose phenotype suggested a likely taxonomic misidentification, and one sample of uncertain provenance. The final set of samples in downstream analyses comprised 328 foxtail millet and 12 green foxtail samples (Supplemental Table S1).

The ‘populations’ module was rerun on the 328 foxtail millet samples without any filtering parameters applied. We subsequently refer to this as the ‘unfiltered SNP dataset.’ The resulting SNP set was filtered in PLINK using the settings ‐‐geno 0.2 (i.e. excluding sites with genotyping data for fewer than 80% of individuals) and ‐‐maf 0.01 (i.e. requiring a minimum minor allele frequency of 1% for sites to be retained). The data set was then pruned for linkage disequilibrium (LD) between markers using the ‐‐indep option, which prunes based on the variance inflation factor by recursively removing SNPs within a sliding window. We used windows of 50 SNPs, with a stepwise shift of five SNPs in length and a variance inflation factor threshold of 2. We subsequently refer to the output from this as the ‘pruned SNP dataset.’ The unfiltered and pruned SNP datasets were used for downstream analyses.

### 2.3 Genetic diversity, population substructure, and differentiation

Species‐ and population‐level diversity statistics were estimated using the Stacks ‘populations’ module on the unfiltered SNP datasets. The ADMIXTURE allocations below were used to define populations within foxtail millet for this analysis.

To examine population structure of the 328 foxtail millet samples, ADMIXTURE (Alexander, Shringarpure, Novembre, & Lange, 2015) was run on the pruned SNP dataset. The model was run for population numbers (*K*) of 1 to 10 with 10 replicate runs per *K*. We explored the most informative value of *K* using the cross‐validation method of Alexander et al. (2015) and by using rubyCorrSieve ver. 1‐7.0 (Campana, Hunt, Jones, & White, 2011) and custom scripts (https://github.com/campanam/ADMIXTURE-stats) to calculate the Δ*K* statistic (Evanno, Regnaut, & Goudet, 2005) and correlate *Q* matrices among multiple runs.

Following selection of the most meaningful value of *K*, the 328 foxtail millet samples were assigned to populations for downstream analysis if they had at least 80% membership of a population according to the ADMIXTURE results. Principal components analyses (PCAs) were carried out in PLINK using the pruned SNP dataset.

### 2.4 Phylogeny

Maximum likelihood trees were constructed for the pruned SNP datasets for both the foxtail millet‐only and foxtail millet and green foxtail combined data sets. Trees were constructed in RAxML (Stamatakis, 2014), selecting the best tree from a rapid bootstrap of 100 replications followed by a maximum likelihood search under the GAMMA rate heterogeneity model with the Lewis correction for ascertainment bias. This search strategy is recommended by the RAxML manual as a reasonable default for SNP datasets based on simulations (Leaché, Banbury, Felsenstein, de Oca, & Stamatakis, 2015).

### 2.5 Phylogenetic analysis incorporating green foxtail accessions from previously published data

Previously published short‐read archive data from GBS of a geographically diverse set of over 200 green foxtail accessions (hereafter referred to as the HKSV set; Huang et al., 2014) were downloaded from NCBI's Bioproject repository (ref. PRJNA243973). Samples in this GBS study were double digested with *Pst*I and *Msp*I, but we anticipated some overlap in sequence with our experiments from tags at the *Pst*I cut sites. The paired‐end raw fastq files were split using a custom script based on the barcoded adaptors on the *Pst*I restriction enzyme‐cut end of the double‐digested DNA samples. Fastq files from the 36 Eurasian accessions in this data set were then used for further analysis with the aim of integrating them with our de novo sample set. Reads from these samples were processed using the ‘process_radtags’ module in Stacks version 1.40 and aligned to the foxtail millet genome using bowtie2 as above. Six HKSV samples were excluded following these steps, as they did not meet filtering and alignment thresholds. The 30 resulting aligned‐read files were combined with aligned‐read files for 328 foxtail millet and 12 green foxtail accessions produced in this study. These were subjected to Stacks pipeline ‘ref_map’ with a minimum stack depth of three. Following initial analyses, four of the HKSV samples (HKSV_CHN1, HKSV_CHN17, HKSV_DEU7, and HKSV_IRAN4) were excluded because they had >30% missing data, and a further five samples were excluded because the original germplasm accession codes showed they matched green foxtail accessions sequenced in our own GBS libraries. The ‘populations’ module was then run on the remaining 361 samples and the output was filtered in PLINK using three settings: ‐‐geno 0.1, ‐‐geno 0.05, and geno ‐‐0.02, with ‐‐maf 0.01 in each case. Lower‐stringency filtering among these three resulted in a matrix with higher amounts of missing data because of the different protocols used to generate the two initial data sets. This data set was pruned for LD as above, and the output was used as input in a PCA implemented using the ade4 package (Chessel, Dufour, & Thioulouse, 2004; Dray & Dufour, 2007; Dray, Dufour, & Chessel, 2007) in R and to construct a maximum likelihood tree in RAxML (Stamatakis, 2014), as above. We report the output from the least‐stringent filtering option (‐‐geno 0.1) because the internal topology of the foxtail millet section of the phylogeny was the most similar to that in analyses without the HKSV samples.

### 2.6 Signatures of selection

To search for genetic markers most strongly differentiating the inferred populations of foxtail millet accessions, BayeScan 2.1 (Foll & Gaggiotti, 2008) was employed. This program uses a Bayesian model to detect the most divergent loci between populations. It is appropriate for the differently sized populations, as it does not introduce bias when smaller sample sizes are used. BayeScan was used to make all six possible pairwise comparisons of the four populations (see Results section) identified by the ADMIXTURE analyses. However, a stricter threshold (at least 90% membership) was used for assigning accessions to populations, meaning that 253 accessions were analyzed here. BayeScan was run for 5,000 iterations with 20 pilot runs, a thinning interval size of 10, 5,000 pilot runs, a burn‐in length of 50,000, and prior odds set to 10. At 18,564 loci, our SNP dataset was relatively small in size and the populations overlapped geographically. These two factors limit the capacity of BayeScan to detect significant outlier loci. Therefore, we used an fixation index (*F*_ST_) percentage cutoff threshold for outliers rather than using the default posterior odds threshold with a false discovery rate of 0.05 (Foll & Gaggiotti, 2008) but set the *F*_ST_ cutoff to a relatively stringent value of 0.05% of markers in each analysis. Fixation index cutoffs of 1 or 0.1% have been applied for studies similarly concerning groups with potential geographical overlap (Cassin‐Sackett, Callicrate, & Fleischer, 2019; Stankowski, Sobel, & Streisfeld, 2017). This criterion was only valid if the distribution of *F*_ST_ results was tail‐like on one or both ends (highest *F*_ST_ values for positive selection or lowest *F*_ST_ values for balancing selection [Foll, 2012]).

To look for evidence of adaptive selection within the domesticated gene pool of foxtail millet, we reviewed the literature to generate a list of genes involved in traits associated with four key environmental adaptations to altitude and latitude as well as the functional domains that characterize these genes (listed in Table 1). Following the BayeScan analysis, all the gene models situated up to 25 kb either side of each identified outlier marker were scanned for annotations with these keywords to identify functional loci that might be associated with environmental adaptation. The genome was scanned using the JBrowse function in Phytozome v.12 on the foxtail millet Yugu 1 genome v 2.2 (Bennetzen et al., 2012; NCBI PRJNA32913), from which functional annotations were retrieved.

**TABLE 1:** Key words for traits associated with environmental adaptations to high altitude and different latitude identified by a literature review and used to search annotations of the *S. italica* cultivar Yugu 1 genome v. 2.2

Traits associated with environmental adaptation	Key words	References
Flowering time	Pseudoresponse regulator PRR, PEPB, CONSTANS (COL)‐like, CCT domain, circadian clock, EARLY FLOWERING, flowering time (FT), CRYPTOCHROME	Cockram et al. (2012) (foxtail millet); Mauro‐Herrera et al. (2013) (foxtail millet); Cho, Yoon, and An (2017) (review)
Phytohormone upregulation—defense against biotic and abiotic stresses	Cytokinins (CK), abscisic acid (ABA), salicylic acid (SA), jasmonate, gibberellins (GA), brassinosteroids (BR)	Ha, Vankova, Yamaguchi‐Shinozaki, Shinozaki, and Tran (2012); Khan, Fatma, Per, Anjum, and Khan (2015); Qi et al. (2018); Vanhaelewyn, Prinsen, Van Der Straeten, and Vandenbussche (2016)
Resistance to higher levels of UV radiation	UV resistance, UVR8, WRKY89, heat shock protein	Brown et al. (2005); Chen et al. (2012); Murakami et al. (2004)
Dwarf phenotype	DELLA, DWARF, gibberellin	Hedden, 2003; Wang, Zhao, Lu, and Deng (2017)

## 3 RESULTS

### 3.1 Species‐level diversity

Following removal of samples with high levels (>30%) of missing data, a total of 124,785 loci containing 118,324 SNPs was retained from the ‘ref_map’ module for the combined foxtail millet (*n* = 328) and green foxtail (*n* = 12) dataset. Overall differentiation between the two species, measured by the *F*_ST_, was 0.083, indicating low overall differentiation. The distribution of *F*_ST_ for individual SNPs was strongly bimodal (Supplemental Figure S2), with 1,955 SNPs having an *F*_ST_ of 1, indicating fixed between‐species differences.

For the foxtail‐millet‐only sample set, 120,550 loci were retained from the ‘ref_map’ module, featuring 107,449 SNPs (the unfiltered SNP dataset). Their distribution on the nine major scaffolds, corresponding to the nine chromosomes, of the foxtail millet genome is shown in Supplemental Figure S3. The SNP density is lowest around the presumed centromere region of each chromosome.

Species‐level diversity estimates taking into account both variant and invariant sites were π = 0.0024 and expected heterozygosity (*H*_E_)  = 0.0021 for foxtail millet and π = 0.0031 and *H*_E _= 0.0028 for green foxtail, indicating that green foxtail harbors somewhat more within‐species variability than foxtail millet.

Filtering for sites genotyped for ≥80% of individuals and minor allele frequency ≥1% retained 18,564 SNPs for the foxtail‐millet‐only sample set. Pruning for LD resulted in 5,677 informative SNPs in the combined foxtail millet and green foxtail dataset and 5,020 SNPs in the foxtail‐millet‐only dataset (the pruned SNP dataset).

### 3.2 Population structure and phylogeny

**FIGURE 1:**
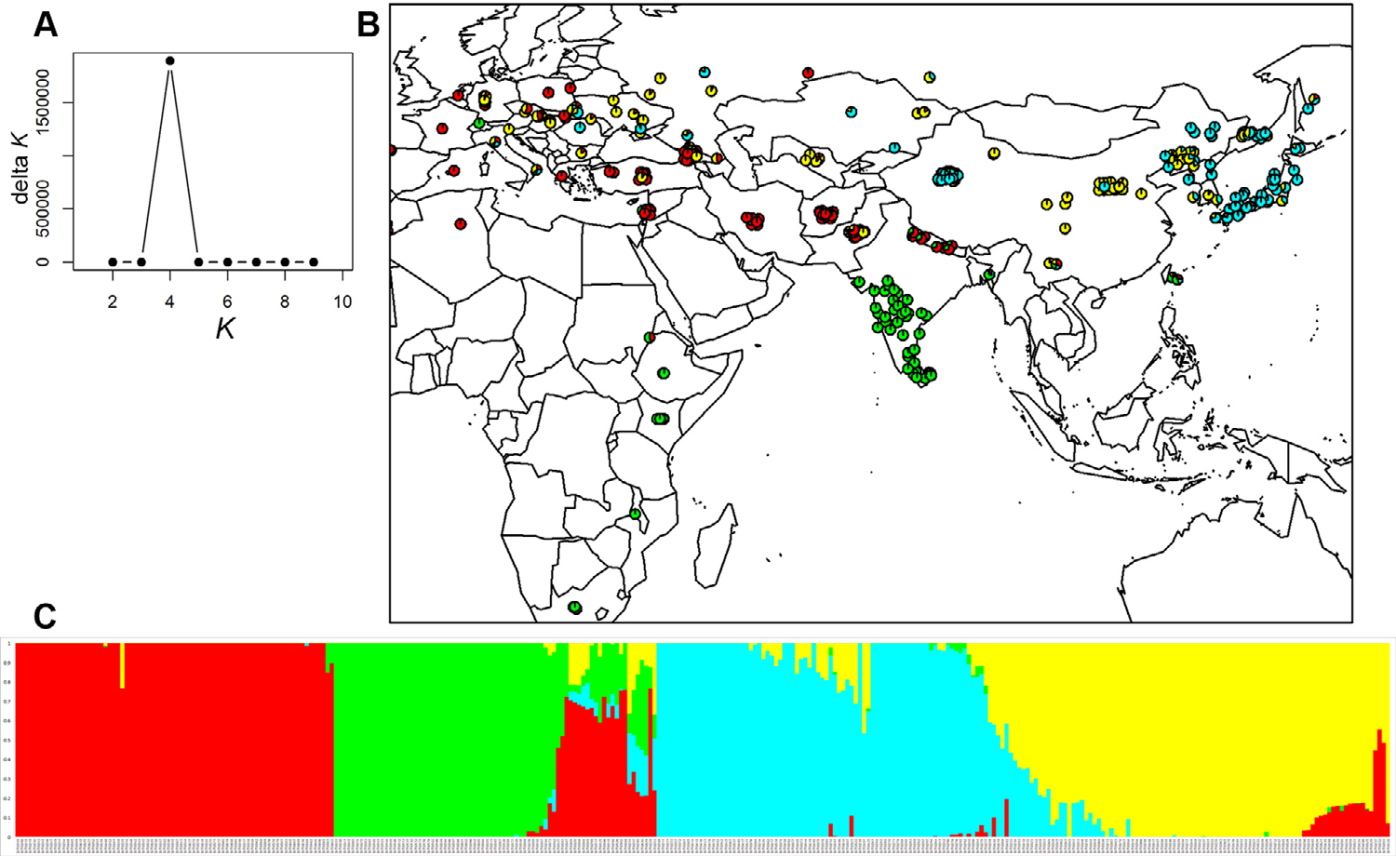
(A) Δ*K* statistic, calculated according to Evanno et al. (2005), plotted against the number of modeled gene pools (*K*) in ADMIXTURE for foxtail millet. (B) Proportional allocations of each of 328 foxtail millet samples to *K* ancestral gene pools inferred using ADMIXTURE (Alexander et al., 2015), under *K =* 4, shown according to sample geographic origin. (C) Proportional allocations of each of 328 foxtail millet samples to *K* ancestral gene pools inferred using ADMIXTURE (Alexander et al., 2015), under *K =* 4, shown as a bar plot

From the ADMIXTURE clustering analysis of genetic structure within foxtail millet, we selected a subpopulation number of *K =* 4 for further analysis, as this model showed highest stability between replicate runs under the *Q*‐matrix correlation criterion in CorrSieve and a clear peak for Δ*K* (Figure 1A). The *Q‐*matrix output for *K* = 4, indicating proportional membership of each accession to the four subpopulations identified, is shown with samples mapped geographically (Figure 1B) and as a bar plot (Figure 1C). Under this model, one cluster (#1, red) dominates southern Asia (Nepal, Pakistan, Afghanistan, Iran, Turkey, and the Near East) and extends west into western and northern Europe and a single sample in north Africa, with a geographical outlier in the southern Ural Mountains. A second cluster (#2, cyan) has a strong Far East focus (including Japan, North and South Korea, and the region of Russia bordering the Sea of Japan) but is also prevalent in samples from western China and found sporadically westward to eastern Europe. The third cluster (#3, green) comprises all samples from India, Sri Lanka, Bangladesh, and eastern and southern Africa with very little admixture, with one geographical outlier from Switzerland. The fourth cluster (#4, yellow) has a widespread east–west distribution further north in Eurasia, from eastern Europe through central Asia to central and northeastern China, with some samples from the Russian Far East also belonging to this group.

**FIGURE 2:**
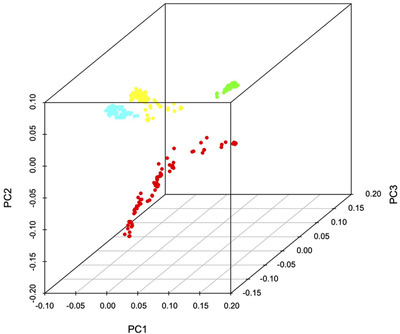
Principal components analysis of 328 foxtail millet samples based on 5,020 single nucleotide polymorphisms with samples colored according to the four gene pools from ADMIXTURE output (sample majority allocation). The axes represent the first three principal components (PCs)

**FIGURE 3:**
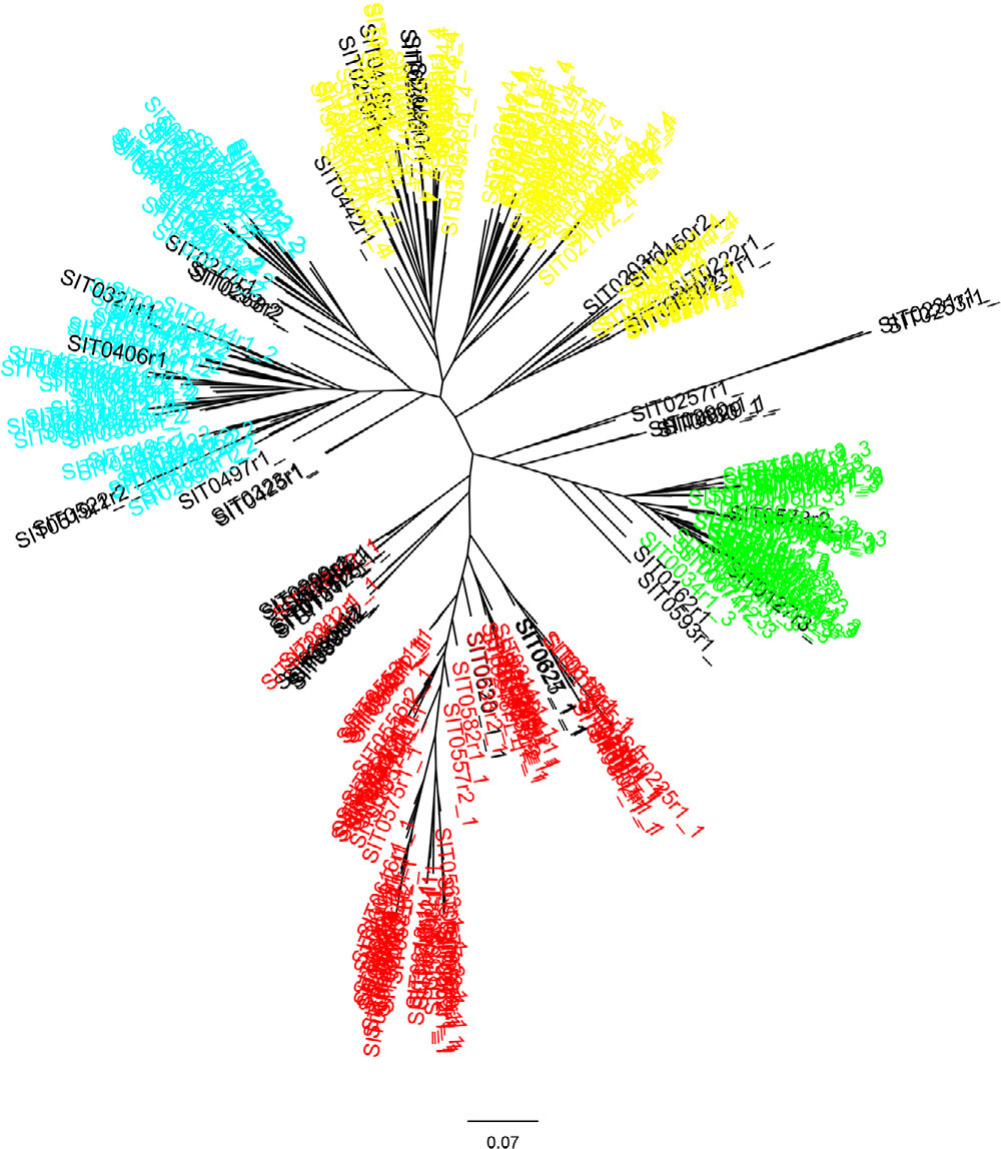
Best unrooted tree found by RAxML (Stamatakis, 2014) using a search strategy of rapid bootstrap followed by a maximum likelihood search under the GAMMA rate heterogeneity model, with the Lewis correction for ascertainment bias. Tree shows phylogenetic relationship between 328 foxtail millet individuals, with samples colored according to the four gene pools from ADMIXTURE output (sample majority allocation)

The four groups identified by ADMIXTURE are supported by results of the PCA (Figure 2) and maximum likelihood trees (Figure 3). Thirteen percent of the variance was explained by the first principal component, 11.9% by the second, and 7.5% by the third principal component (Figure 2). Consistent with other genetic diversity measures (see below), the red cluster was the most widely distributed through PC space and the green cluster the most tightly cohesive.

**FIGURE 4:**
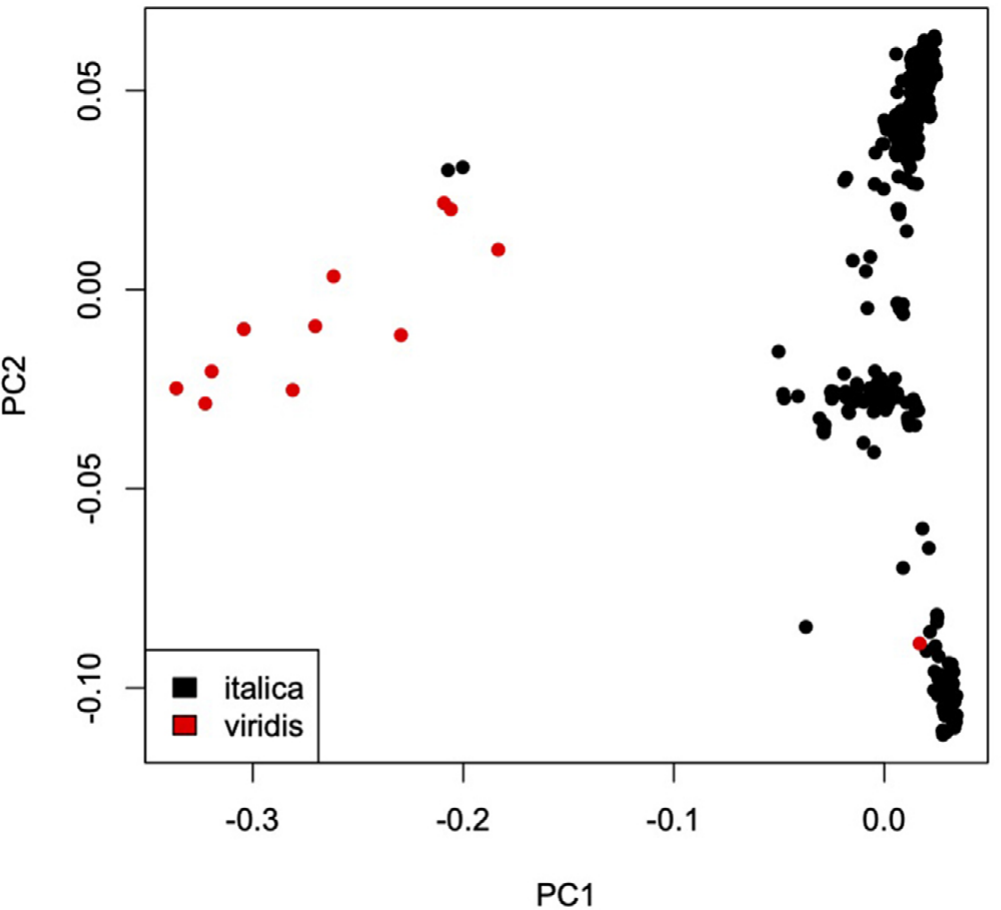
Principal components analysis of 328 foxtail millet and 12 green foxtail samples based on 5,677 single nucleotide polymorphisms. The axes represent the first two principal components (PCs)

**FIGURE 5:**
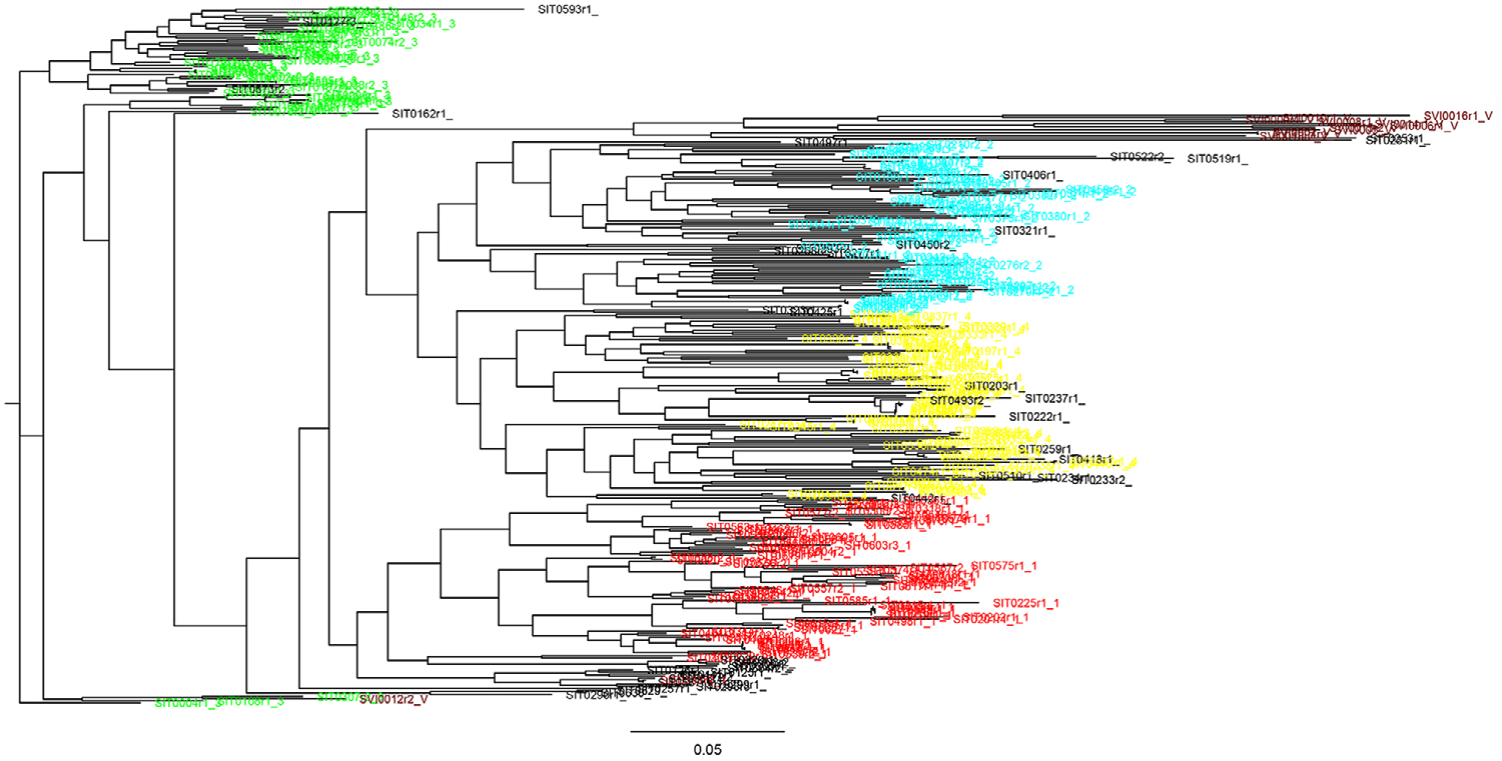
Best tree found by RAxML (Stamatakis, 2014) using a search strategy of rapid bootstrap followed by a maximum likelihood search under the GAMMA rate heterogeneity model, with the Lewis correction for ascertainment bias. Tree shows phylogenetic relationship between 328 foxtail millet and 12 green foxtail individuals, with samples colored according to the four gene pools from ADMIXTURE output (sample majority allocation) for foxtail millet (red, yellow, green, cyan groups) and brown for green foxtail. The main green foxtail clade was set as the outgroup

When the 12 green foxtail samples were included in the PCA, a similar amount of variance was explained by the first two principal components: 13.7 and 11.0% for PC1 and PC2, respectively. The first principal component mainly separated foxtail millet from green foxtail samples, while the second principal component largely explained differentiation within foxtail millet (Figure 4). The PCA suggested that two samples, classified as foxtail millet (SIT0231 and SIT0253), were in fact green foxtail, and one sample thought to be green foxtail (SVI0012) should be identified as foxtail millet. These likely misidentifications were also supported by the clustering of samples in the maximum likelihood tree including green foxtail samples (Figure 5). With this tree rooted on the main green foxtail cluster, all groups of foxtail millet emerged as monophyletic relative to green foxtail. This relationship was explored further with the inclusion of additional green foxtail samples from an independent dataset (below).

### 3.3 Clustering and phylogenetic analysis incorporating additional green foxtail samples

**FIGURE 6:**
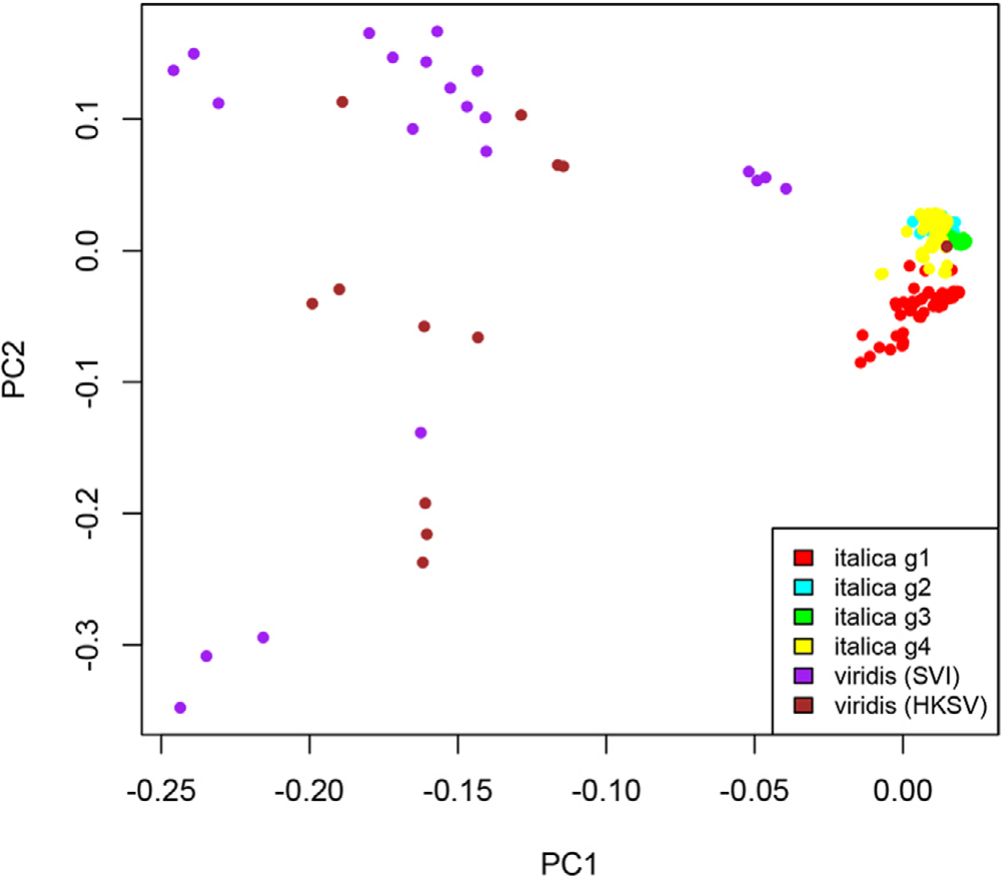
Principal components analysis of 328 foxtail millet (SIT), 12 green foxtail (SVI), and 21 additional green foxtail from Huang et al., 2014 (HKSV) samples based on 4,515 SNPs. Samples colored according to the four gene pools from ADMIXTURE output (foxtail millet, red, yellow, green, cyan clusters); brown, SVI; purple, HKSV samples. The axes represent the first two principal components (PCs)

**FIGURE 7:**
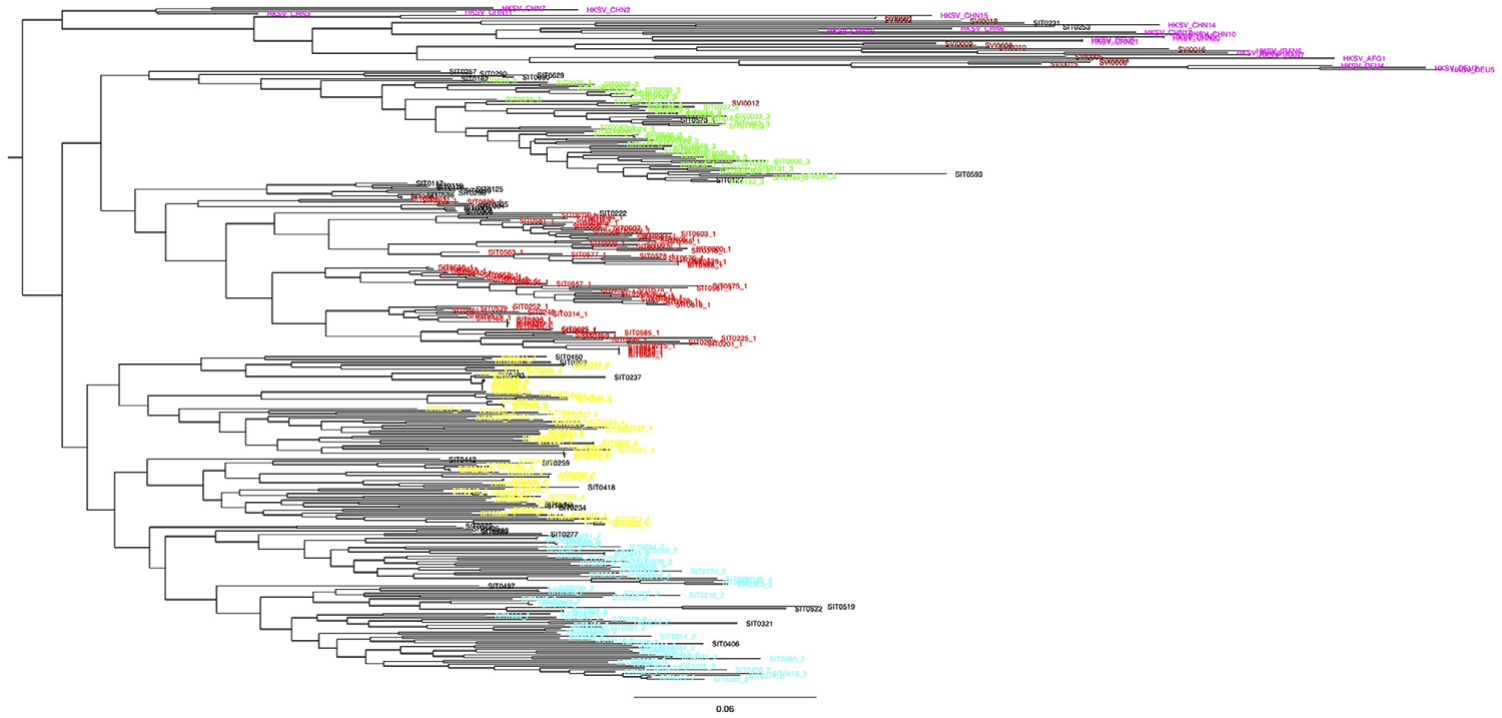
Best tree found by RAxML (Stamatakis, 2014) using a search strategy of rapid bootstrap followed by a maximum likelihood search under the GAMMA rate heterogeneity model, with the Lewis correction for ascertainment bias. Tree shows phylogenetic relationship between 328 foxtail millet individuals, with samples colored according to the four gene pools from ADMIXTURE output (foxtail millet), or brown (green foxtail, SVI) or purple (additional green foxtail from Huang et al., 2014, HKSV). The tree was rooted on the green foxtail clade (mixed SVI/HKSV)

After inclusion of GBS datasets from 21 previously published green foxtail accessions (Huang et al., 2014), the number of SNPs using the three filtering settings ‐‐geno 0.1, ‐‐geno 0.05, and geno ‐‐0.02, after LD pruning, were 4,515; 2,719; and 825, respectively. Although the least‐stringent (‐‐geno 0.1) setting produced a matrix with large amounts of missing data, especially for the HKSV samples, we report results from this setting as the tree topology for the foxtail millet samples was most similar to that reported above (Figures 3 and 5). A scatterplot of the first two principal components (Figure 6) shows that these axes accounted for 20.2 and 9.9% of the total variance, respectively. The foxtail millet accessions cluster tightly and are separated from the much more widely scattered HKSV and green foxtail samples. The latter two groups are not obviously separated. In the phylogeny (Figure 7), virtually all the HKSV and green foxtail samples form a sister clade to all the foxtail millet samples. Within this green foxtail clade, the majority of the green foxtail samples, which originated outside China, cluster with the non‐Chinese HKSV samples (from southern and southwestern Asia and Europe), while the Chinese HKSV samples form a separate group. Rooting the foxtail millet samples on the green foxtail clade shows that the former constitutes a near‐monophyletic cluster, with no subpopulation of foxtail millet obviously more ancestral than any other.

### 3.4 Population genetic diversity and differentiation

Measures of genetic diversity for the four foxtail millet clusters identified by ADMIXTURE are shown in Supplemental Table S4. Cluster 1 (red in Figure 1) shows the highest genetic diversity by a variety of measures and Cluster 3 (green) is clearly the least diverse.

Pairwise *F*_ST_ between the four clusters is shown in Supplemental Table S5, with ranges between 0.131 and 0.212 for the six pairwise comparisons. The least‐differentiated populations are Cluster 2 (cyan) and Cluster 4 (yellow). Cluster 3 (green) shows the highest average differentiation from every other population.

### 3.5 Signatures of selection on candidate adaptive genes

Because of the wide geographical range across which foxtail millet has been cultivated, it has been selected for local or regional adaptation to a wide range of agricultural environments. To explore possible genomic regions underlying this adaptation, plots of the BayeScan results, which aim to detect the most divergent loci between populations, reveal potential outlier loci for all six pairwise comparisons of the four populations identified. Positive selection is more likely in four of the comparisons and balancing selection in the remaining two comparisons (Supplemental Table S6; Supplemental Figure S7). The top 0.05% of SNPs with the highest (or lowest) *F*_ST_ values equates to nine SNPs per analysis. Two of these SNPs were hits in multiple comparisons, such that a total of 52 significant SNPs were detected in total. In the 50‐kb windows spanning these SNPs, a total of nine candidate genes with annotations matching keywords for genes controlling potential environmental adaptations to altitude and latitude were revealed (Supplemental Table S8). For the comparison of both Groups 1 and 4 as well as Groups 2 and 3, a locus scaffold_ 9_43137022 was in close proximity to both *Seita.9G371300.1* (a heat shock protein gene) and *Seita.9G371000.1* (a jasmonate ZIM domain protein gene). This outlier is under apparent balancing selection in the former and positive selection in the latter case. The second locus of potential interest is situated at position scaffold_8_688223 and was discovered in the comparisons of Groups 1 vs. 2 as well as Groups 3 vs. 4. Two genes (*Seita.8G012100.1* and *Seita.8G012000.1*) encoding Phototropic‐responsive NPH3 family proteins are found in the search window. These are predicted to be involved in the blue light signaling pathway. For outlier loci at positions scaffold_4_532966, scaffold_7_2816053, and scaffold_1_28247088, three genes encoding thioredoxin superfamily proteins were discovered in close proximity. These were: *Seita.4G008600.1* and *Seita.7G279700.1* (comparison of Groups 1 vs. 2) and *Seita.1G203000.1* (comparison of Groups 2 vs. 4). Finally, a gene (*Seita.5G124300*) encoding a brassinosteroid was found near locus scaffold_5_10426326 in the comparison of Groups 1 vs. 2, and an oxygenase superfamily protein gene (*Seita.4G045400*) near locus scaffold_4_3360999 in the comparison of Groups 2 vs. 4.

## 4 DISCUSSION

### 4.1 Nucleotide diversity in foxtail millet and green foxtail

The raw number of SNPs in foxtail millet (118,324) called here across 328 samples was approximately one‐third that in the one previous GBS analysis of a global foxtail millet set of 181 samples (Upadhyaya et al., 2015). The ∼50% fewer samples in their study had a less geographically balanced distribution, but they used the restriction enzyme *ApeK*I, which is expected to produce many more tags than *Pst*I. This study retained a similar number (∼17,770) of high‐quality SNPs for downstream analysis after filtering.

We found a high level of nucleotide diversity in foxtail millet, with the estimated value of π = 0.0024, ∼2.5‐fold higher than reported by Jia et al. (2013). Reduced‐representation library sequencing approaches, such as restriction‐site‐associated DNA sequencing and GBS typically underestimate genetic diversity relative to whole‐genome sequencing because of base composition heterogeneity and allele dropout at restriction sites (Cariou, Duret, & Charlat, 2016). Nevertheless, the higher GBS‐based nucleotide diversity observed here compared with previous studies likely represents a real result because of the more even geographic distribution of samples in our study and their status as landraces. We did not knowingly include any cultivars, which are likely to show reduced genetic diversity compared with landraces, in our sampling. This finding highlights the value of studying genomic diversity in foxtail millet across its entire range of cultivation as represented by landrace collections to maximize genetic resources for functional genomic studies and breeding in this crop (Hu, Mauro‐Herrera, & Doust, 2018).

The wild species, green foxtail, showed higher genetic diversity measures even with small sample size (*n* = 12) than the domesticated foxtail millet, as is typically the case for crops that have experienced a gradual reduction in genetic diversity as a result of a series of founder effects and selection after domestication (Allaby, Ware, & Kistler, 2019). Crop wild relatives are often poorly represented in germplasm collections (Hunt, Badakshi, Howe, Jones, & Heslop‐Harrison, 2014). Thanks to recent collecting efforts, representative collections of green foxtail now exist for both North America (Huang & Feldman, 2017) and China (Jia et al., 2013). Future collection efforts spanning Eurasia would be worthwhile to understand the cross‐continental population genomics of this global weed and explore genetic introgression of green foxtail into foxtail millet.

### 4.2 Genetic structure of foxtail millet

Previous population genetic or genomic studies in foxtail millet have explored the structuring of genetic variation, principally in relation to intra‐Chinese geography and sowing time (Jia et al., 2013; Wang et al., 2012) and races (morphologically distinct complexes), which also differ in geographical distribution of their cultivation and show distinct flowering time responses under particular day length conditions (Prasada Rao, de Wet, Brink, & Mengesha, 1987; Upadhyaya et al., 2015). Wang et al. (2012) identified three genetic groups among 250 Chinese landraces that showed distinct geographical distributions and sowing times. Jia et al. (2013) found the 916 accessions in their study divided into two groups: spring‐sown types from northeastern China and high‐altitude areas of northwestern China, and summer‐sown types largely from central and southern China. The distributions of our Clusters 2 (cyan) and 4 (yellow) within China could plausibly correspond to Jia et al. (2013)'s spring‐sown and summer‐sown groups, respectively.

Upadhyaya et al. (2015) found six genetic clusters, with two comprised of accessions belonging to races *maxima* and *moharica* (not clearly separated) and four clusters containing accessions belonging to race *indica*, but did not directly discuss the geographical distribution of each cluster. We did not distinguish samples in our study based on subspecific taxonomy, as information on races was available only for some of the samples. From its distribution, our Cluster 3 (green) probably corresponds to race *indica* as identified morphologically by Prasada Rao et al. (1987) and genetically by Upadhyaya et al. (2015). The geographical distributions of races *maxima* and *moharica* as described by Prasada Rao et al. (1987) do not appear to correspond to those of any of the remaining three clusters we observed, supporting the finding of Upadhyaya et al. (2015) that these morphotypes do not constitute genetically distinct groupings likely because of inherent phenotypic plasticity in the species. Although our Cluster 1 (red) occupies a geographical range fitting the descriptions of *maxima* and *moharica*, our phylogenetic and ADMIXTURE analyses indicate that this group is more closely related to Cluster 3 (green) than to Clusters 2 or 4.

### 4.3 Support for a monophyletic origin of cultivated foxtail millet

The phylogenetic tree of these 12 green foxtail accessions in relation to the foxtail millet samples indicated that the two species are mutually monophyletic (Figure 5), with the PCA (Figure 4) reinforcing their clear genetic separation. The between‐species *F*_ST_ of 0.083 indicates low overall differentiation, although *F*_ST_ is sensitive to the approach for calling SNPs (Bhatia, Patterson, Sankararaman, & Price, 2013). In prairie sunflower (*Helianthus petiolaris* Nutt.), the incipient stages of speciation between ecotypes that diverged ∼10,000 years ago, that is, a similar likely timeframe to the divergence between foxtail millet and green foxtail, involved divergence of only a few large genomic regions with relatively low overall genome‐wide F*_ST_* (Andrew & Rieseberg, 2013). Because correct inference of monophyly of a given taxon is strongly dependent on adequate sampling of both outgroup and ingroup taxa, we explored the validity of combining green foxtail sequence data from two independent GBS studies to increase the sample size and geographical representation of green foxtail in the resulting phylogeny. Foxtail millet remained monophyletic in relation to green foxtail when samples from an independent GBS study were integrated into our analyses (Figure 7), increasing the robustness of this finding.

Our results suggested that the combination of the two datasets in the Stacks pipeline was valid, with HKSV and green foxtail samples mixed in genetic space rather than forming distinct clusters, which might have suggested a primary effect of library protocol. In the phylogenetic analysis, the samples grouped according to geographical origin within Eurasia, supporting the biological meaningfulness of the dataset. Although the total number of SNPs in the combined dataset was small, and missing data levels were heterogeneous and high for some subsets, the resulting phylogeny was very similar to that with only green foxtail and foxtail millet samples included. It has previously been shown (Tripp, Tsai, Zhuang, & Dexter, 2017) that phylogenetic reconstruction based on restriction‐site‐associated DNA sequencing data can be robust to the presence of high levels of missing data.

Huang et al. (2014) identified three genetic groups within green foxtail distributed in both North America and Eurasia. One group, focused in China, was genetically cohesive with the small number (*n* = 16) of foxtail millet accessions successfully sequenced in that study consistent with a single domestication center of foxtail millet in northern China. The monophyly of foxtail millet relative to the HKSV and green foxtail clade in our tree also accords with a single origin, but because of the small number of green foxtail relative to foxtail millet samples in our analyses, we were not able to identify genetic groups within the wild species. However, in the PCA (Figure 6), four green foxtail samples lie much closer to the foxtail millet samples than the rest; these four are from geographically diverse regions of China. Further sampling and analyses should explore whether the genetic similarity of these green foxtail samples with foxtail millet reflects ancestry, postdomestication gene flow, or is an artefact of the dataset.

### 4.4 Geographic expansion of foxtail millet integrating genetic and archaeobotanical data

The green foxtail root is basal to the split between the southern and western (red and green) clusters and pan‐northern‐Eurasian, including Chinese (yellow and cyan clusters). In a simple scenario of single domestication followed by expansion from northern China, it would be expected that the red and green clusters occupy a derived position relative to the yellow and cyan clusters in the tree. Today, the red cluster is entirely absent from China, instead characterizing populations from Nepal, Pakistan, and Afghanistan. The earliest known appearance of foxtail millet in this broad region was at the start of the second millennium BC from northern India (Stevens et al., 2016), at least 3,500 yr later than its initial domestication in northern China. Either the southern and western Asia populations of foxtail millet may have incorporated additional genetic input from local green foxtail that has confounded the phylogeny or this cluster formerly extended further to the north and east into China but has now been totally replaced by other genotypes there.

The westward extension of the red cluster through southwestern Asia (Iran, Syria, the Caucasus, and Turkey) suggests a route of expansion from east to west along this routeway, eventually reaching Europe. Archaeobotanical data trace foxtail millet to the early second millennium BC in the Caucasus (Herrscher et al., 2018) and the later part of the same millennium in both Turkey (Longford, Drinnan, & Sagona, 2009; Miller, 2010; Pasternak, 1998) and Greece (Kroll, 1983, 1991). The green cluster, confined to India, Bangladesh, and sub‐Saharan East Africa, most plausibly evolved from the red cluster and involved a genetic bottleneck associated with a founder event. Foxtail millet spread into southern India from the late second millennium BC following its appearance in the northern part of the country, reaching Sri Lanka by ∼175 BC (Pokharia, Kharakwal, & Srivastava, 2014; Stevens et al., 2016). It is present at Qasr Ibrim in lower Egypt from the middle of the first millennium BC (Clapham & Rowley‐Conwy, 2007), but there is little or no archaeobotanical record of its southward spread in Africa.

In Europe, the red cluster likely represents one of two introduction episodes of foxtail millet, with the yellow and blue clusters reaching Europe via a more northerly route. Currently there is no coherent chronology for this expansion, as a number of records in Europe appear to predate those in central Asia by two to three millennia, but questions remain over both the dating and identification of some of these early finds in Europe (Hunt et al., 2008). The weakly differentiated cyan and yellow clusters also show substantial geographical overlap, although Japan, where foxtail appears from the late or final Jomon (first half of the first millennium BC; Nasu & Momohara, 2016) is dominated by the cyan group. Unlike in northeastern China, where foxtail millet is spring sown, the related genotypes in Japan are summer sown throughout most of the country (https://www.pref.aichi.jp/nogyo-keiei/nogyo-aichi/tukuchaou/awakibi/index.html, last accessed 30 Apr. 2020). This observation warrants further investigation into flowering time response in different regions.

### 4.5 Evidence for adaptive selection between foxtail millet genetic groups

We explored our dataset to test for signatures of selection that might reflect environmental adaptations that evolved and were selected for in the spread of foxtail millet west, south, and east from China. The ability to detect genetic loci implicated in differential selection was limited by the reduced representation library sequencing approach chosen and by the relatively sparse functional annotation of the foxtail millet genome at present. Nevertheless, we identified a number of putative genes with predicted functions of potential relevance to environmental adaptation in the vicinity of the most differentiated SNP loci. One of the nine SNPs (scaffold_9_43137022) lies within the putative gene itself; the others are adjacent to the genes and could lie in promoter regions for these. Regarding gene functions, the jasmonate ZIM domain protein (Seita.9G371000.1) may aid in abiotic stress resistance; homologous proteins play a role in resistance against pathogens in sugarcane (F. Liu et al., 2017), which is closely related to *Setaria*. The thioredoxin genes encode enzymes for antioxidants and are important signaling compounds on the interface of plant stress and physiological responses (Arnér & Holmgren, 2000; Gelhaye, Rouhier, Navrot, & Jacquot, 2005), potentially enabling acclimation. *Seita.4G045400* encodes a 2‐oxoglutarate/Fe(II)‐dependent dioxygenase, a relatively uncommon enzyme (only 11 genes in foxtail millet), which is one of the most versatile oxidative enzymes in plant physiology, including biosynthesis of metabolites important in abiotic stress such as UV tolerance (Farrow & Facchini, 2014). *Seita.5G124300.1* encodes a brassinosteroid, and the brassinosteroid pathway is known to play a role in maintaining plant growth under conditions of environmental stress (Planas‐Riverola et al., 2019). More thorough investigation of the genome for selective sweeps as well as expression studies and phenotypic analysis would demonstrate whether the genes identified here have in fact played a role in environmental adaptation.

In conclusion, the genetic structure of foxtail millet corresponds strongly with geography across Asia, Europe, and Africa, likely representing the spread of different subpopulations from a single origin in China. Whole‐genome sequencing is the requisite next step to determine the extent of wild introgression and to test hypothesized adaptive plant stress responses in different geographical regions.

## AUTHOR CONTRIBUTIONS

Harriet V. Hunt: Conceptualization, Methodology, Formal Analysis, Investigation, Data Curation, Writing (Original Draft); Natalia A.S. Przelomska: Methodology, Formal Analysis, Investigation, Data Curation, Writing (Original Draft); Michael G. Campana: Software, Formal Analysis, Writing (Review & Editing), James Cockram: Writing (Review & Editing), Supervision; H. Frances J. Bligh: Writing (Review & Editing), Supervision; Catherine J. Kneale: Investigation; Olga I. Romanova: Resources, Writing (Review & Editing); Elena V. Malinovskaya: Resources; Martin K. Jones: Writing (Review & Editing), Supervision, Project Administration.

## CONFLICT OF INTEREST

The authors declare that they have no conflict of interest.

## Supporting information

Supplemental Material

Supplemental Material

Supplemental Material

Supplemental Material

Supplemental Material

Supplemental Material

## Data Availability

Sequence data generated in this study are openly available in GenBank, BioProject ID: PRJNA637841.
