# Supplementary material for: Population genomic structure of Eurasian and African foxtail millet landrace accessions inferred from genotyping‐by‐sequencing
Source: Plant Genome. 2021 Feb 4;14(1):e20081. doi: 10.1002/tpg2.20081 (PMC8638668; doi:10.1002/tpg2.20081)

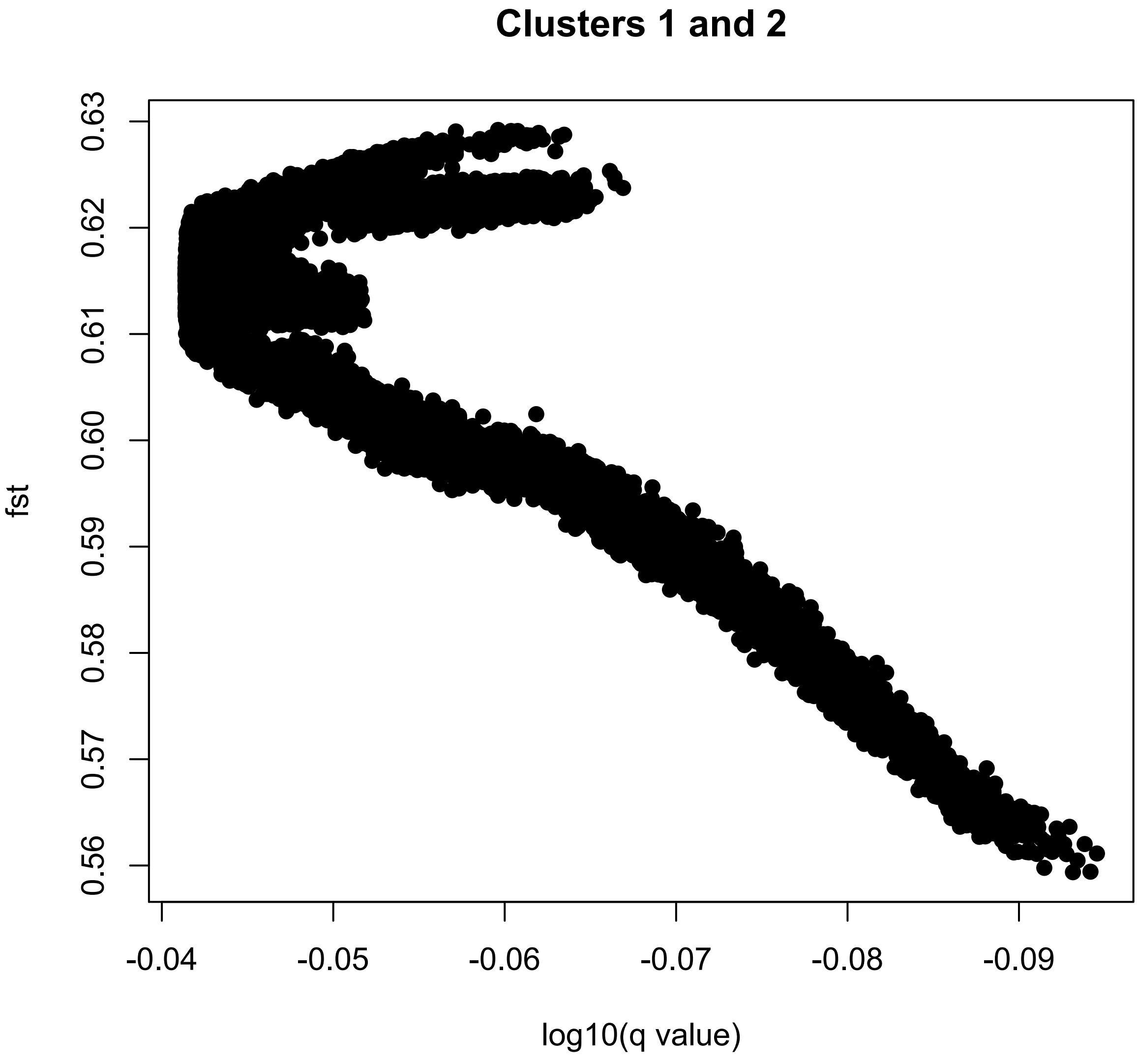

Clusters 1 and 2
fst
0.56
0.57
0.58
0.59
0.60
0.61
0.62
0.63
-0.04
-0.05
-0.06
-0.07
-0.08
-0.09
log10(q value)

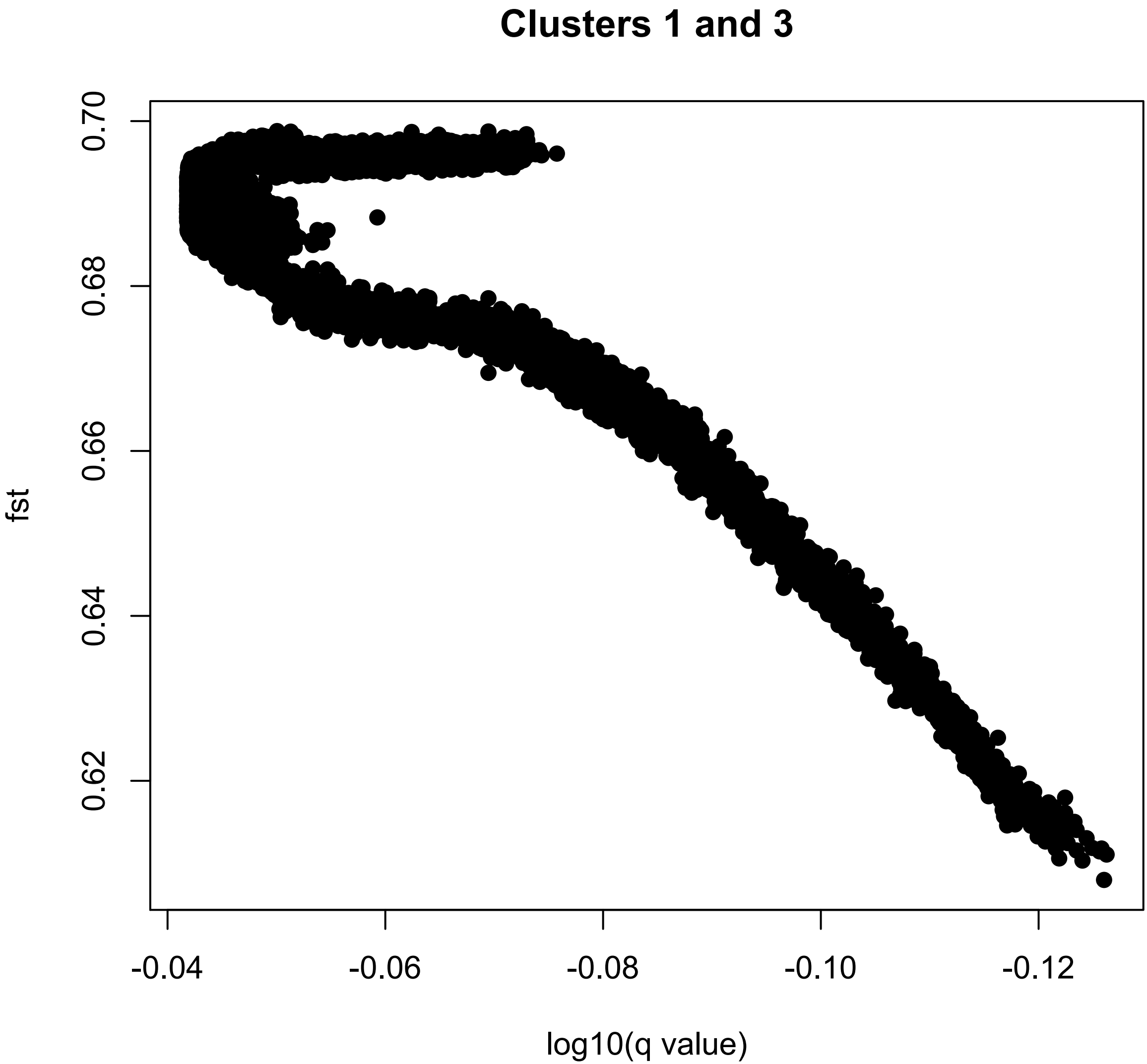
Clusters 1 and 3
fst
0.62
0.64
0.66
0.68
0.70
log10(q value)
-0.04
-0.06
-0.08
-0.10
-0.12

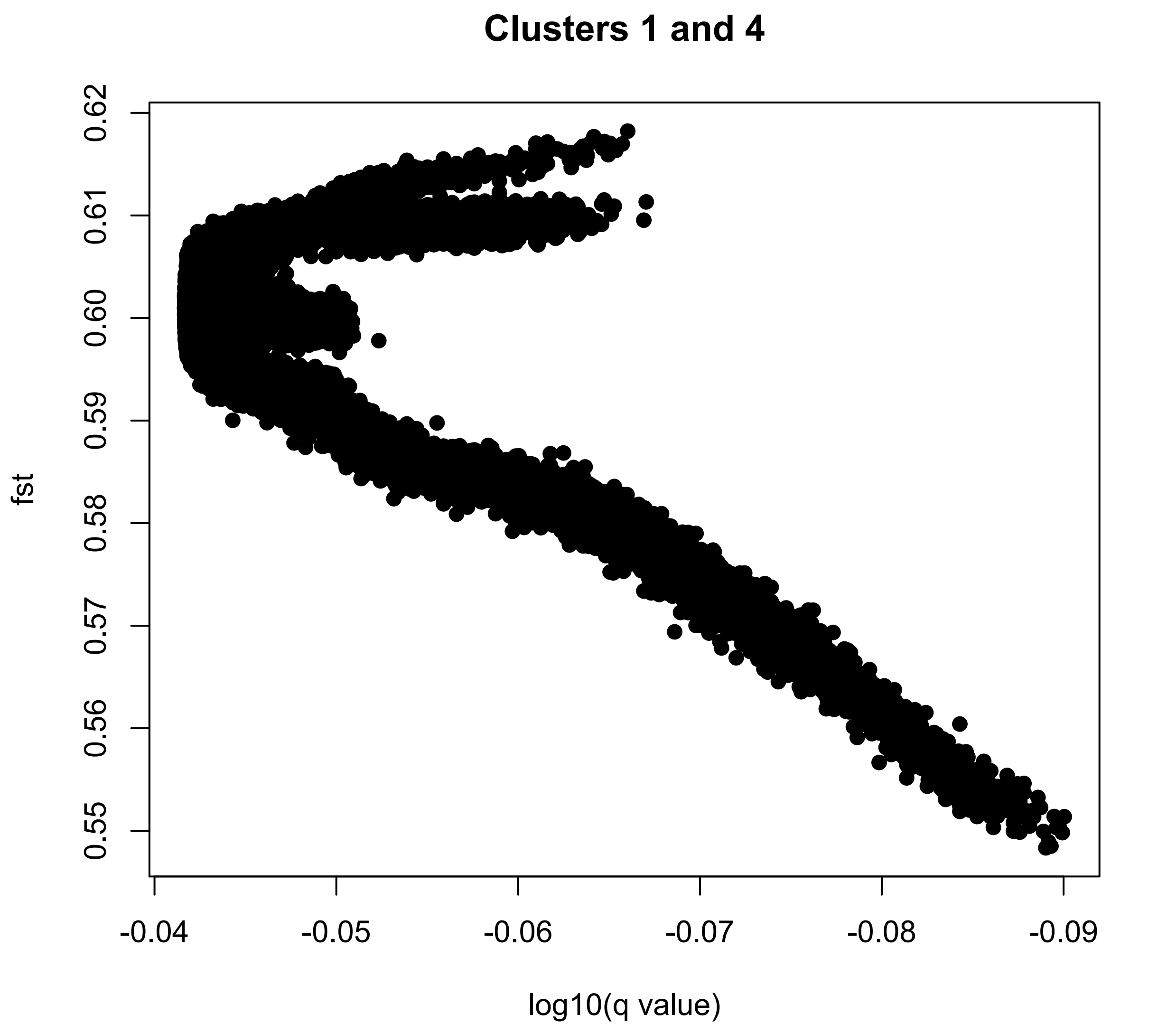
Clusters 1 and 4
fst
0.55
0.56
0.57
0.58
0.59
0.60
0.61
0.62
-0.04
-0.05
-0.06
-0.07
-0.08
-0.09
log10(q value)

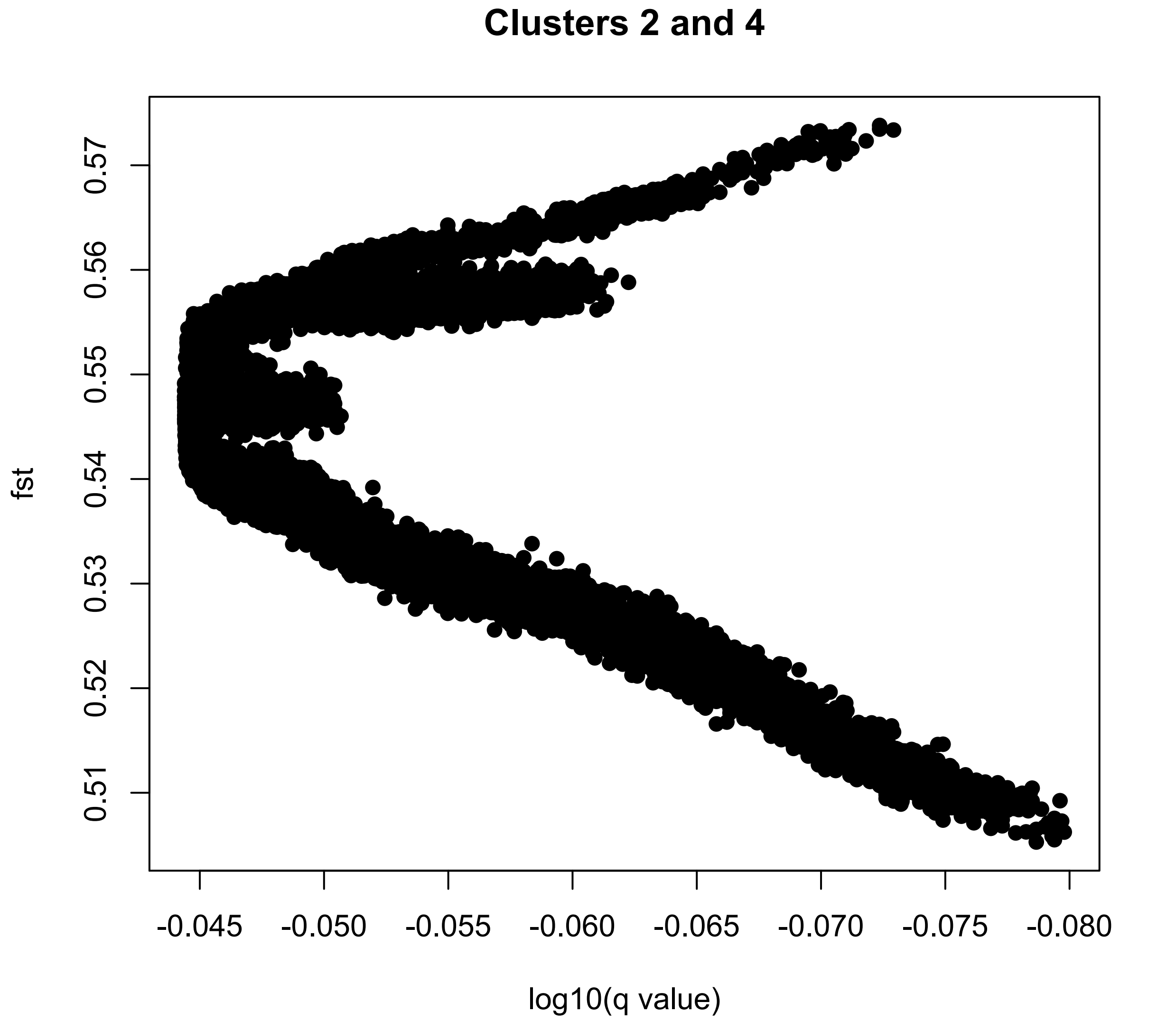
Clusters 2 and 4
fst
0.51
0.52
0.53
0.54
0.55
0.56
0.57
-0.045
-0.050
-0.055
-0.060
-0.065
-0.070
-0.075
-0.080
log10(q value)

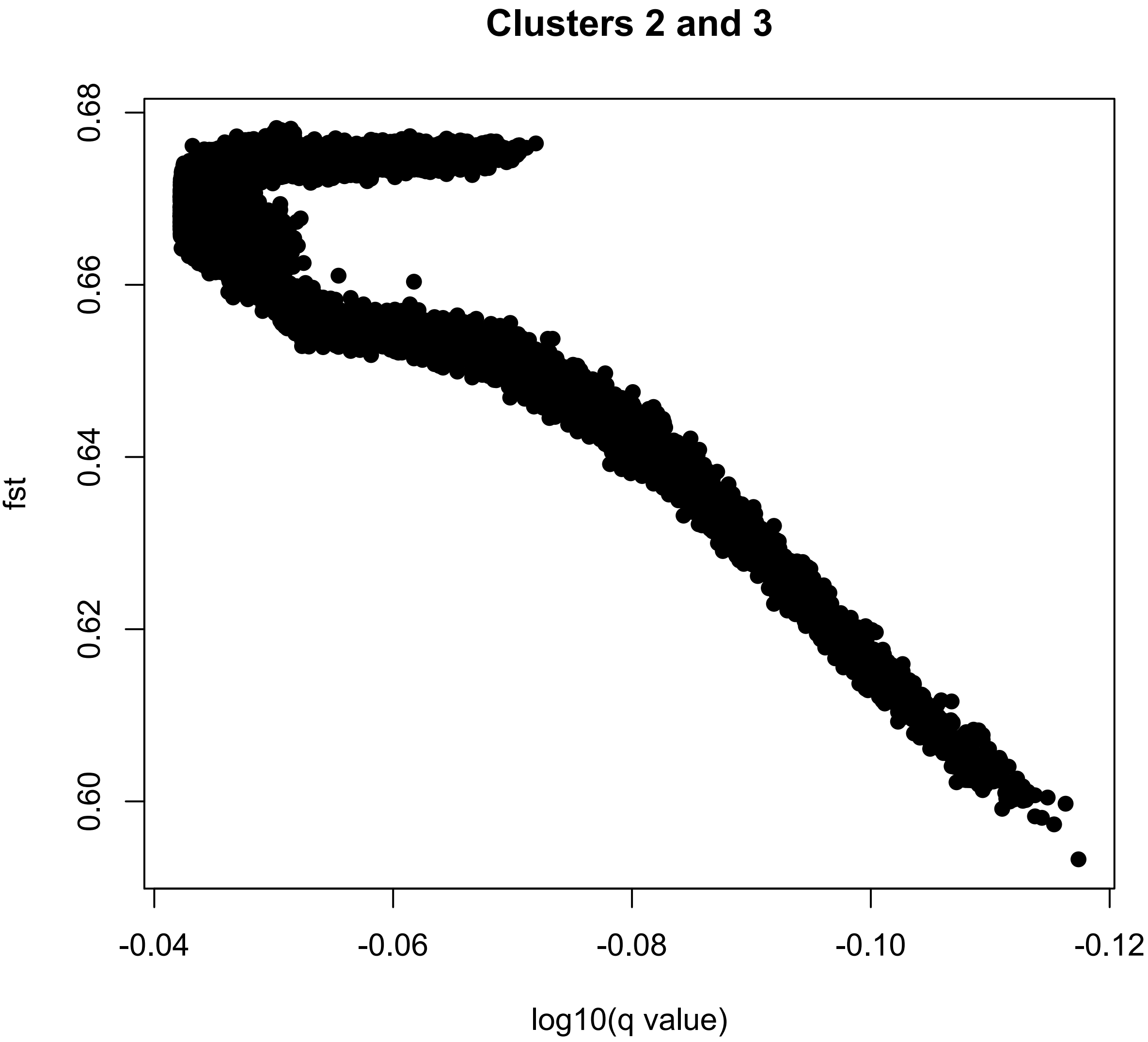
Clusters 2 and 3
fst
0.60
0.62
0.64
0.66
0.68
-0.04
-0.06
-0.08
-0.10
-0.12
log10(q value)

Clusters 3 and 4

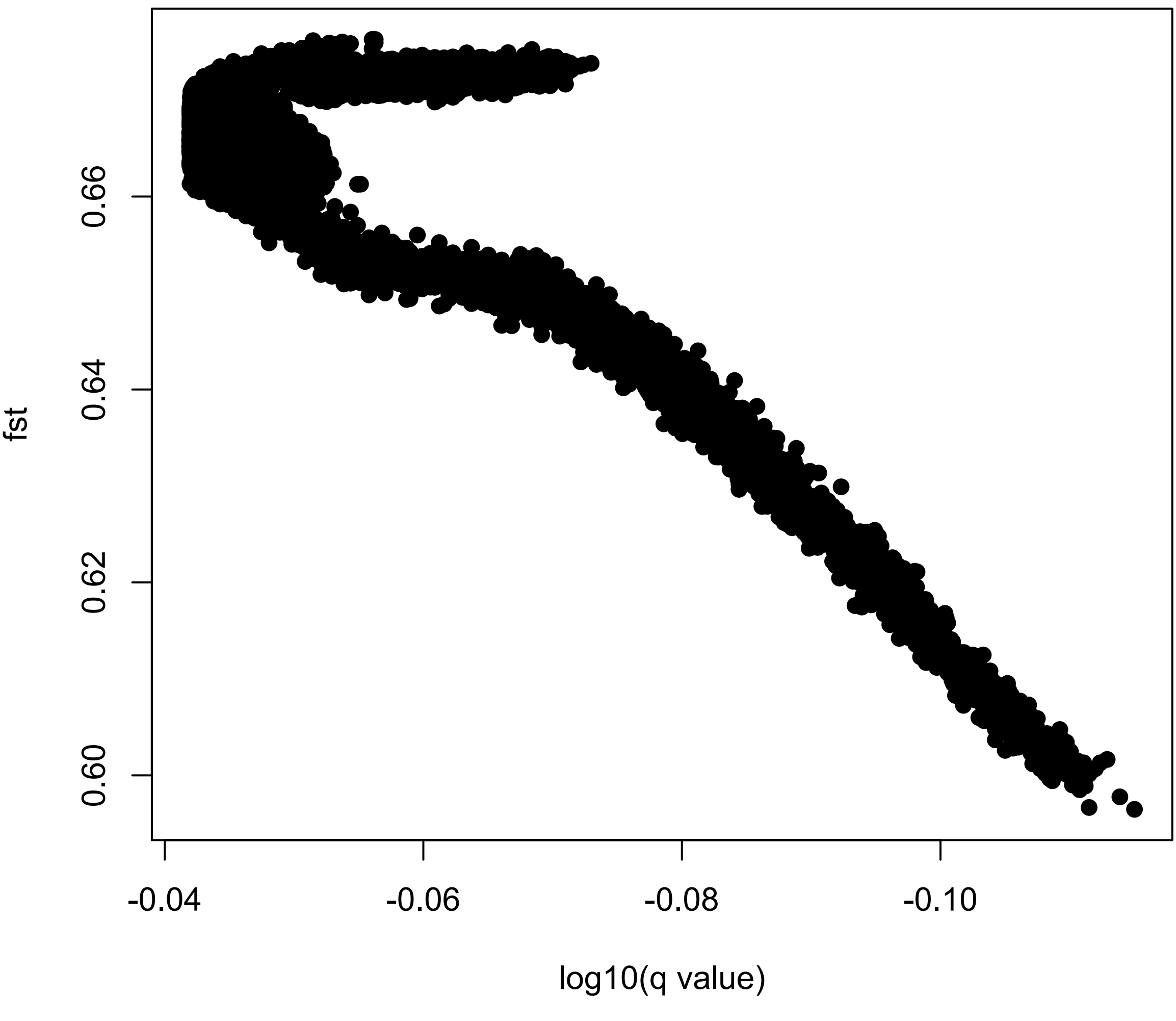

Supplement: Supplementary file 1 [file TPG2-14-e20081-s005.pdf]
