# Supplementary material for: Population genomic structure of Eurasian and African foxtail millet landrace accessions inferred from genotyping‐by‐sequencing
Source: Plant Genome. 2021 Feb 4;14(1):e20081. doi: 10.1002/tpg2.20081 (PMC8638668; doi:10.1002/tpg2.20081)
Supplement: Supplementary file 2 [file TPG2-14-e20081-s001.docx]

| **K4 group** | **K4 group** | **Positive selection (upper tail)** | **Balancing selection (lower tail)** |
| --- | --- | --- | --- |
| 1 | 2 | N | O |
| 1 | 3 | N | O |
| 1 | 4 | O | N |
| 2 | 3 | N | O |
| 2 | 4 | O | N |
| 3 | 4 | N | O |
| O=observed |  |  |  |
| N=not observed |  |  |  |
