# Supplementary figures and images for: Population genomic structure of Eurasian and African foxtail millet landrace accessions inferred from genotyping‐by‐sequencing
Source: Plant Genome. 2021 Feb 4;14(1):e20081. doi: 10.1002/tpg2.20081 (PMC8638668; doi:10.1002/tpg2.20081)

## Clusters 1 and 2

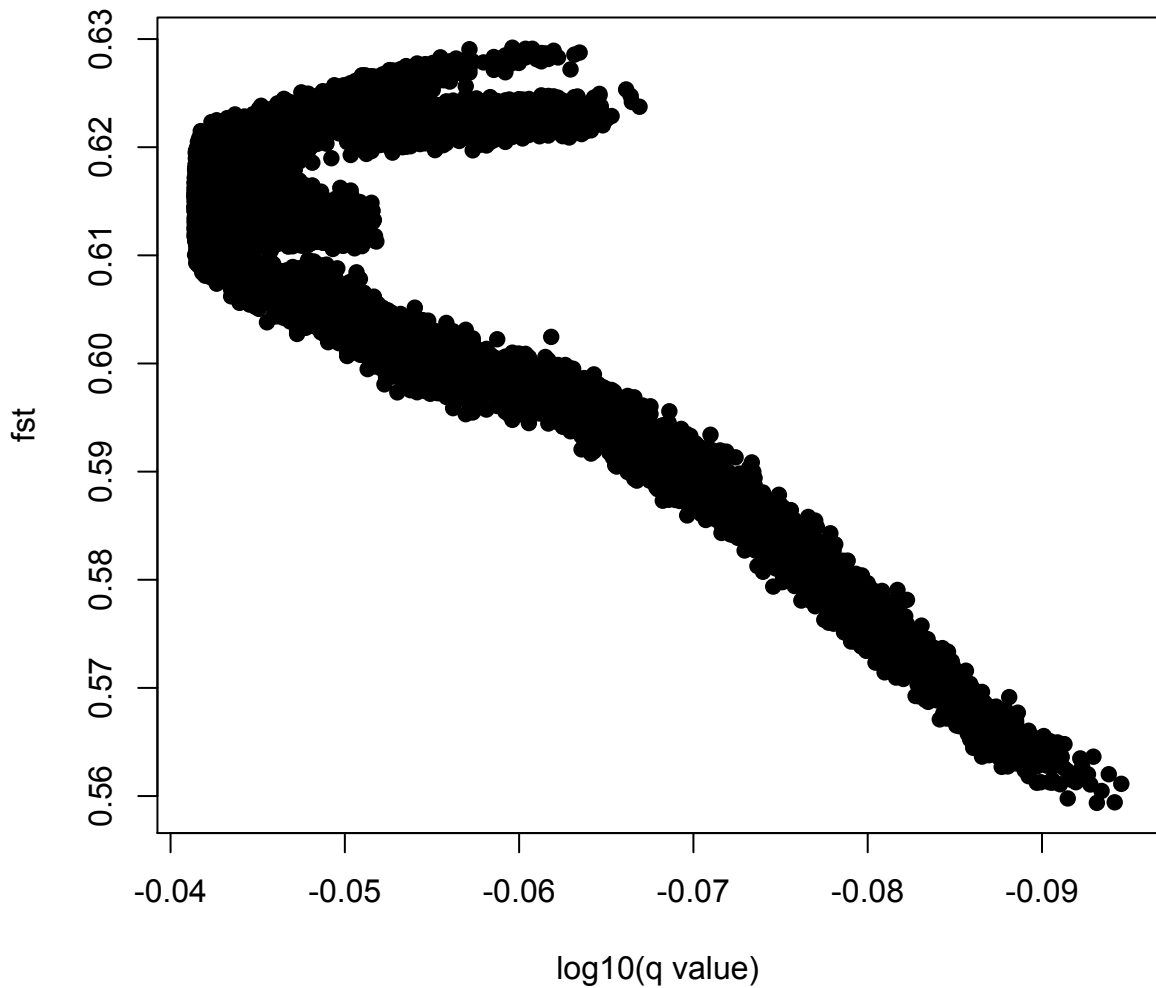

## Clusters 1 and 3

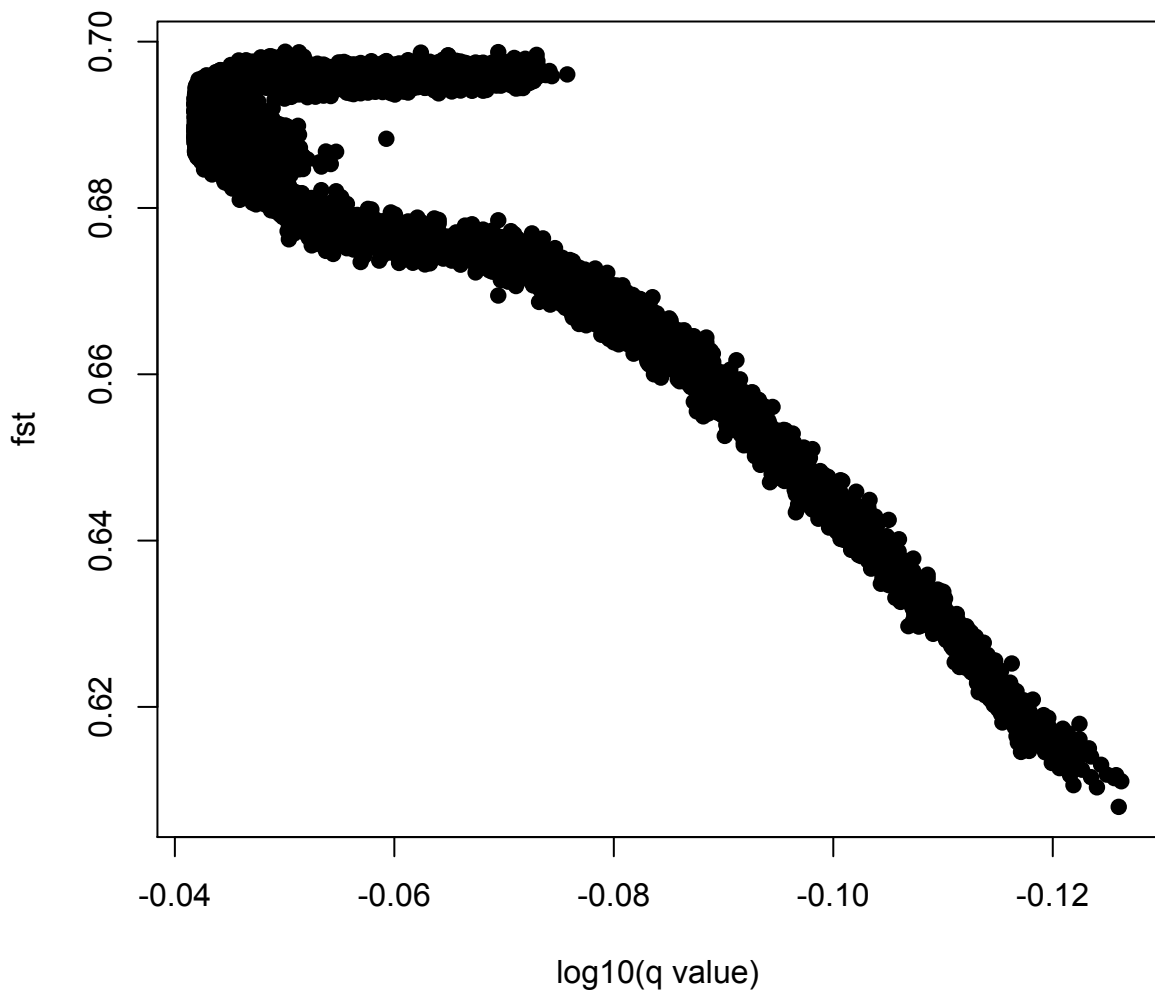

Clusters 1 and 4

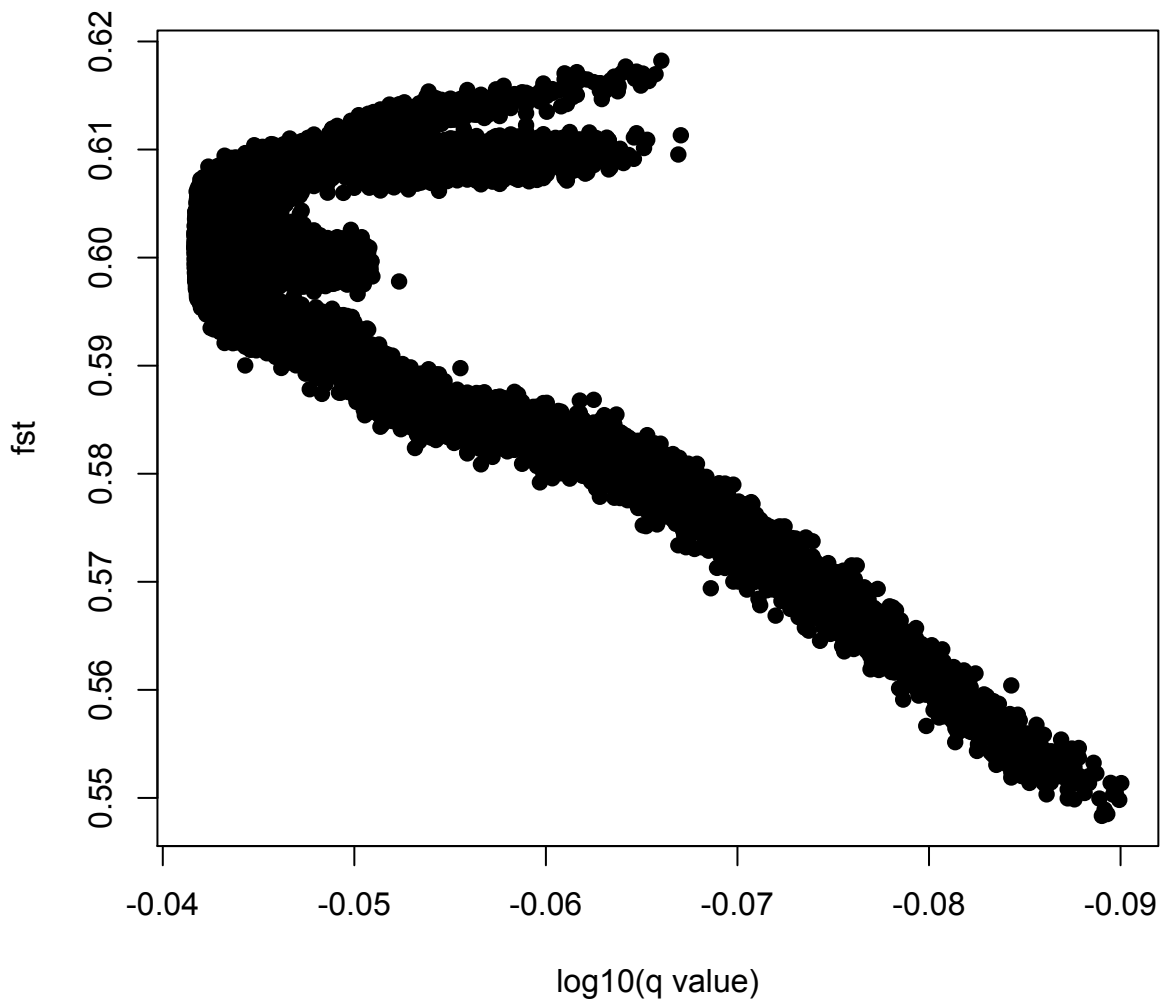

## Clusters 2 and 4

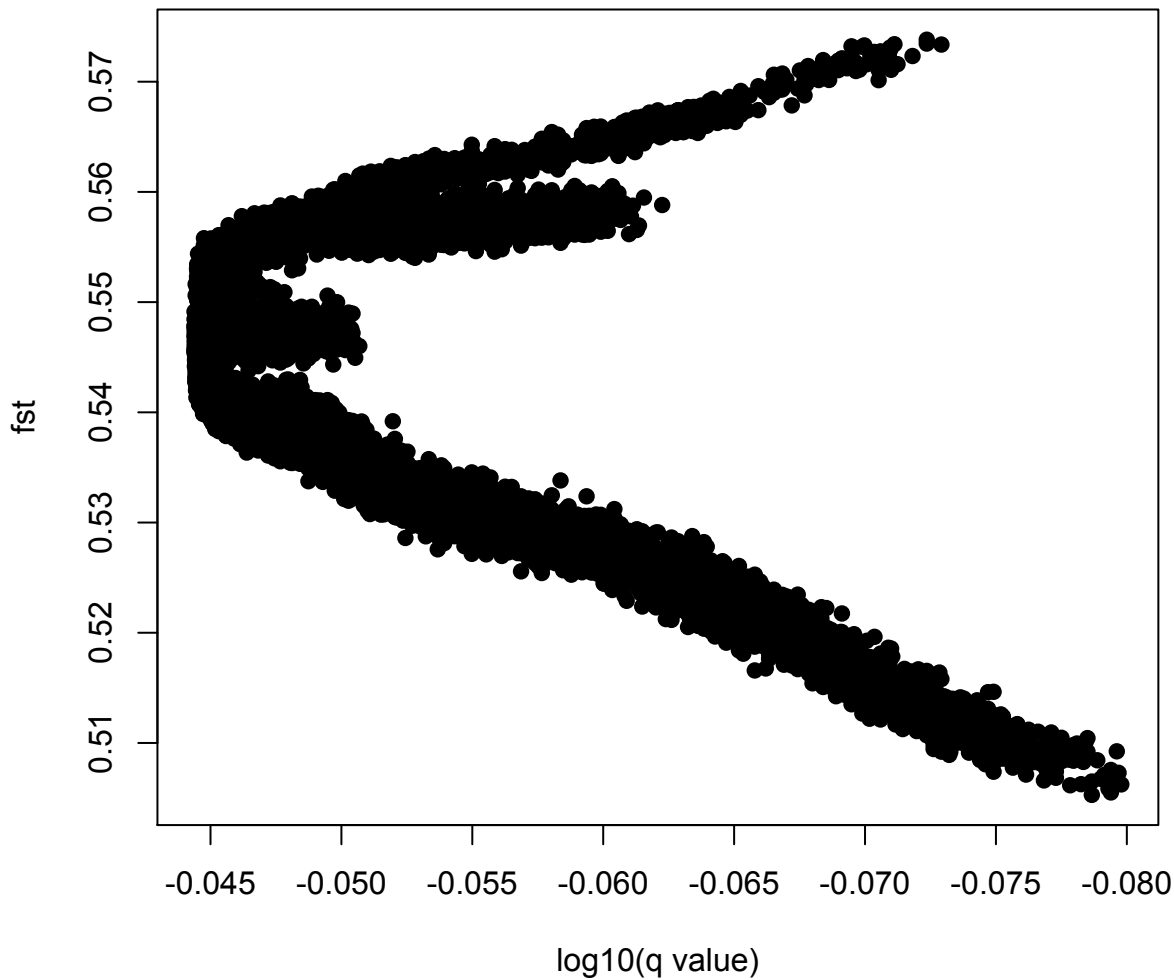

## Clusters 2 and 3

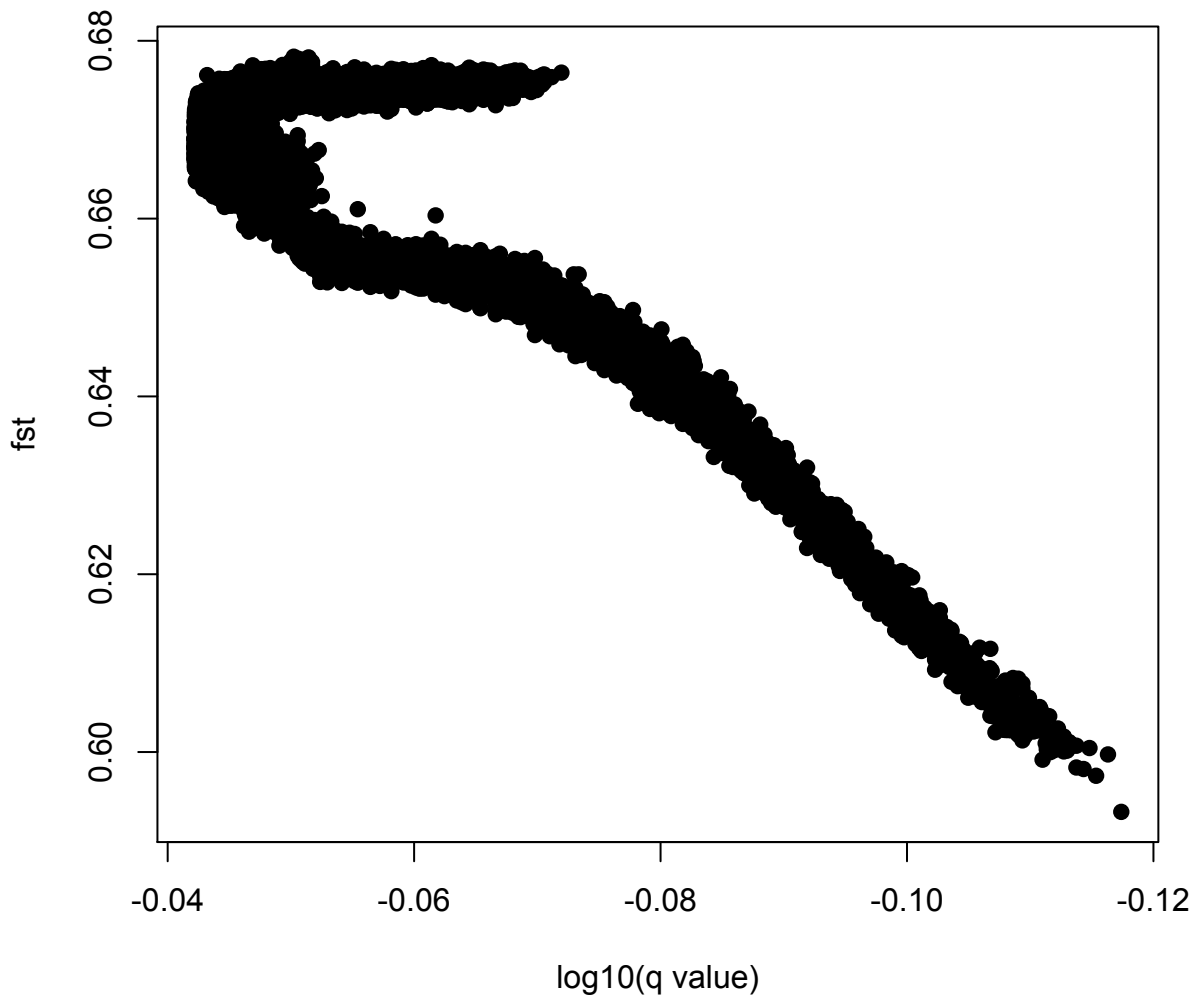

# Clusters 3 and 4

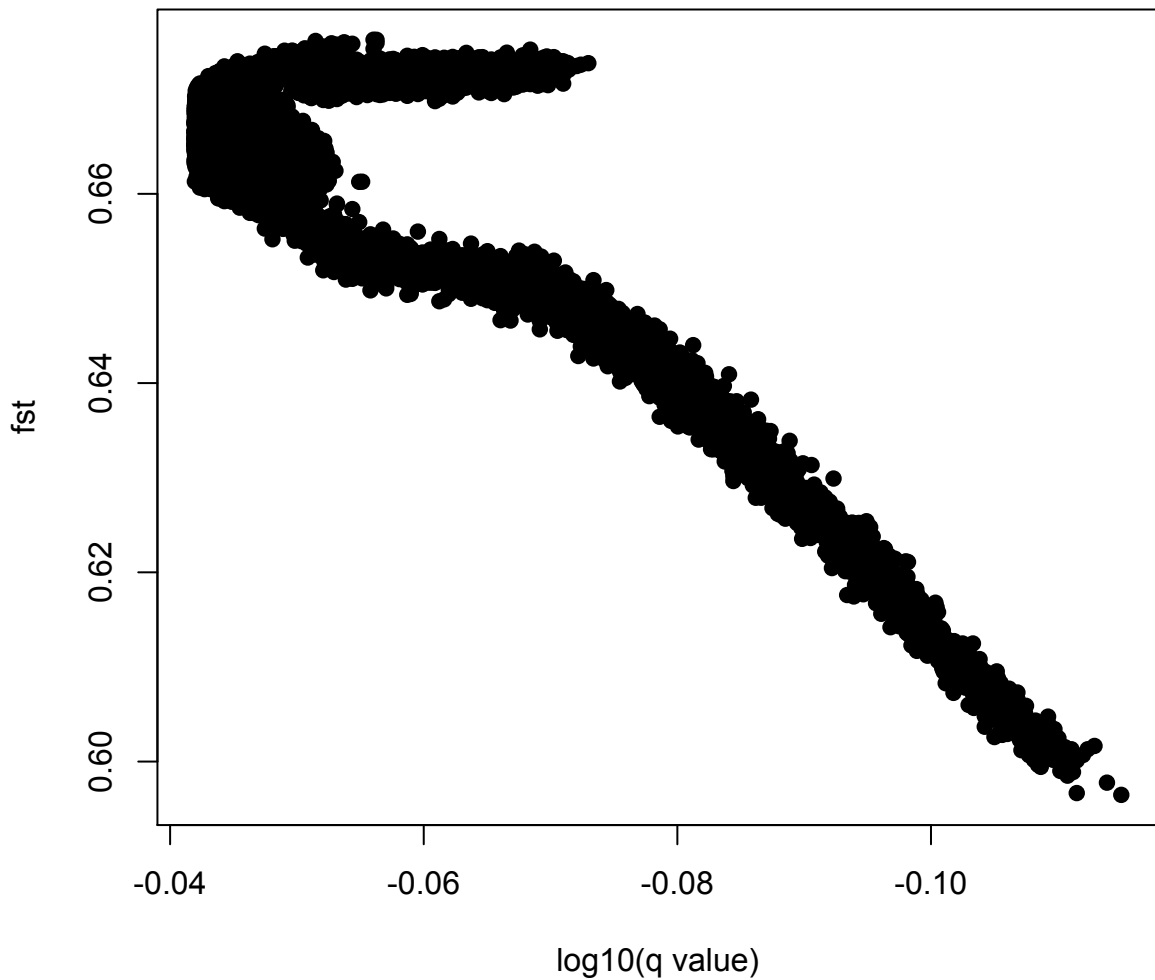

Supplement: Supplementary file 5 — Supplemental Material [file TPG2-14-e20081-s001.pdf]
