# Supplementary material for: Population genomic structure of Eurasian and African foxtail millet landrace accessions inferred from genotyping‐by‐sequencing
Source: Plant Genome. 2021 Feb 4;14(1):e20081. doi: 10.1002/tpg2.20081 (PMC8638668; doi:10.1002/tpg2.20081)
Supplement: Supplementary file 6 — Supplemental Material [file TPG2-14-e20081-s005.docx]

| **Comparison** | **Group 1 and Group 4** |  |  |  |  |  |
| --- | --- | --- | --- | --- | --- | --- |
|  |  |  |  |  |  |  |
| **Chromosome and position** | **Gene** | **Relevant annotation** | **Adjacent gene** | **Relevant annotation** | |  |
| scaffold_2_1843415 | - |  |  |  |  |  |
| scaffold_3_50380846 | Seita.3G405800.1 |  |  |  |  |  |
| scaffold_4_35527122 | Seita.4G233200.1 |  |  |  |  |  |
| scaffold_4_35527261 | Seita.4G233200.1 |  |  |  |  |  |
| scaffold_5_16269871 | - |  |  |  |  |  |
| scaffold_7_22631546 | Seita.7G134000.1 |  |  |  |  |  |
| scaffold_8_32256251 | - |  |  |  |  |  |
| scaffold_8_988002 | Seita.8G017300.1 |  |  |  |  |  |
| scaffold_9_43137022 | Seita.9G371300.1 | heat shock protein 101 | Seita.9G371000.1 | jasmonate-zim-domain protein 1 |  |  |
|  |  |  |  |  |  |  |
| **Comparison** | **Group 2 and Group 4** |  |  |  |  |  |
|  |  |  |  |  |  |  |
| **Chromosome and position** | **Gene** | **Relevant annotation** | **Adjacent gene** | **Relevant annotation** | |  |
| scaffold_1_28247088 | Seita.1G202600.1 |  | Seita.1G203000.1 | thioredoxin |  |  |
| scaffold_3_33554325 | Seita.3G298500.1 |  |  |  |  |  |
| scaffold_3_43312417 | - |  |  |  |  |  |
| scaffold_4_1031741 | Seita.4G015800.1 |  |  |  |  |  |
| scaffold_4_3360999 | - |  | Seita.4G045400.1 | 2OG-Fe(II) oxygenase superfamily | | |
| scaffold_8_34111998 | - |  |  |  |  |  |
| scaffold_8_34112022 | - |  |  |  |  |  |
| scaffold_8_6226908 | Seita.8G064300.1 |  |  |  |  |  |
| scaffold_9_25077786 | Seita.9G276700.1 |  |  |  |  |  |
|  |  |  |  |  |  |  |
| **Comparison** | **Group 3 and Group 4** |  |  |  |  |  |
|  |  |  |  |  |  |  |
| **Chromosome and position** | **Gene** | **Relevant annotation** | **Adjacent gene** | **Relevant annotation** | **Adjacent gene** | **Relevant annotation** |
| scaffold_3_50387755 | Seita.3G405900.1 |  |  |  |  |  |
| scaffold_4_35773413 | Seita.4G235500.1 |  |  |  |  |  |
| scaffold_7_13717630 | Seita.7G046100.1 |  |  |  |  |  |
| scaffold_7_13845696 |  |  |  |  |  |  |
| scaffold_7_13845696 | Seita.7G047000.1 |  |  |  |  |  |
| scaffold_8_34187652 | - |  |  |  |  |  |
| scaffold_8_5655280 | Seita.8G060600.1 |  |  |  |  |  |
| scaffold_8_688223 | Seita.8G011900.1 |  | Seita.8G012100.1 | Phototropic-responsive NPH3 family protein | Seita.8G012000.1 | Phototropic-responsive NPH3 family protein |
| scaffold_8_7810933 | - |  |  |  |  |  |
|  |  |  |  |  |  |  |
| **Comparison** | **Group 2 and Group 3** |  |  |  |  |  |
|  |  |  |  |  |  |  |
| **Chromosome and position** | **Gene** | **Relevant annotation** | **Adjacent gene** |  |  |  |
| scaffold_1_37616871 | Seita.1G315200.1 |  |  |  |  |  |
| scaffold_1_7828938 | - |  |  |  |  |  |
| scaffold_3_2461511 | Seita.3G040500.1 |  |  |  |  |  |
| scaffold_4_881026 | Seita.4G013600.1 |  |  |  |  |  |
| scaffold_6_31696436 | - |  |  |  |  |  |
| scaffold_6_35417239 | Seita.6G246400.1 |  |  |  |  |  |
| scaffold_7_28694508 | Seita.7G219300.1 |  |  |  |  |  |
| scaffold_7_32913483 | Seita.7G281200.1 |  |  |  |  |  |
| scaffold_9_43136985 | Seita.9G371300.1 | heat shock protein 101 | Seita.9G371000.1 | jasmonate-zim-domain protein 1 |  |  |
|  |  |  |  |  |  |  |
| **Comparison** | **Group 1 and Group 3** |  |  |  |  |  |
|  |  |  |  |  |  |  |
| **Chromosome and position** | **Gene** | **Relevant annotation** | **Adjacent gene** |  |  |  |
| scaffold_1_7243071 | - |  |  |  |  |  |
| scaffold_2_34302343 | - |  |  |  |  |  |
| scaffold_2_3740466 | - |  |  |  |  |  |
| scaffold_2_759023 | Seita.2G011900.1 |  |  |  |  |  |
| scaffold_6_5983344 | Seita.6G069500.1 |  |  |  |  |  |
| scaffold_7_13717956 | Seita.7G046100.1 |  |  |  |  |  |
| scaffold_7_17978855 | - |  |  |  |  |  |
| scaffold_8_34187652 | - |  |  |  |  |  |
| scaffold_9_727898 | Seita.9G013800.1 |  |  |  |  |  |
|  |  |  |  |  |  |  |
| **Comparison** | **Group 1 and Group 2** |  |  |  |  |  |
|  |  |  |  |  |  |  |
| **Chromosome and position** | **Gene** | **Relevant annotation** | **Adjacent gene** | **Relevant annotation** | **Adjacent gene** | **Relevant annotation** |
| scaffold_1_14727169 | Seita.1G141400.1 |  |  |  |  |  |
| scaffold_2_34139882 | Seita.2G236900.1 |  |  |  |  |  |
| scaffold_4_532966 | Seita.4G008400.1 |  | Seita.4G008600.1 | thioredoxin |  |  |
| scaffold_5_10426326 | Seita.5G124600.1 |  | Seita.5G124300.1 | brassinosteroid |  |  |
| scaffold_7_2816053 | Seita.7G015400.1 |  | Seita.7G279700.1 | thioredoxin |  |  |
| scaffold_7_2816263 | Seita.7G015400.1 |  |  |  |  |  |
| scaffold_7_32822711 | Seita.7G279800.1 |  |  |  |  |  |
| scaffold_8_6091019 | Seita.8G063300.1 |  |  |  |  |  |
| scaffold_8_688169 | Seita.8G011900.1 |  | Seita.8G012100.1 | Phototropic-responsive NPH3 family protein | Seita.8G012000.1 | Phototropic-responsive NPH3 family protein |
